# High‐Performance Hydrogel‐Encapsulated Engineered Exosomes for Supporting Endoplasmic Reticulum Homeostasis and Boosting Diabetic Bone Regeneration

**DOI:** 10.1002/advs.202309491

**Published:** 2024-02-21

**Authors:** Yulan Liu, Sihan Lin, Zeqian Xu, Yuqiong Wu, Guifang Wang, Guangzheng Yang, Lingyan Cao, Haishuang Chang, Mingliang Zhou, Xinquan Jiang

**Affiliations:** ^1^ Department of Prosthodontics Shanghai Ninth People's Hospital Shanghai Jiao Tong University School of Medicine College of Stomatology Shanghai Jiao Tong University National Center for Stomatology National Clinical Research Center for Oral Diseases Shanghai Key Laboratory of Stomatology Shanghai Research Institute of Stomatology Shanghai Engineering Research Center of Advanced Dental Technology and Materials Shanghai 200125 China; ^2^ Shanghai Institute of Precision Medicine Shanghai Ninth People's Hospital Shanghai Jiaotong University School of Medicine Shanghai 200125 China

**Keywords:** diabetic bone regeneration, endoplasmic reticulum homeostasis, engineered exosome, hydrogel

## Abstract

The regeneration of bone defects in diabetic patients still faces challenges, as the intrinsic healing process is impaired by hyperglycemia. Inspired by the discovery that the endoplasmic reticulum (ER) is in a state of excessive stress and dysfunction under hyperglycemia, leading to osteogenic disorder, a novel engineered exosome is proposed to modulate ER homeostasis for restoring the function of mesenchymal stem cells (MSCs). The results indicate that the constructed engineered exosomes efficiently regulate ER homeostasis and dramatically facilitate the function of MSCs in the hyperglycemic niche. Additionally, the underlying therapeutic mechanism of exosomes is elucidated. The results reveal that exosomes can directly provide recipient cells with SHP2 for the activation of mitophagy and elimination of mtROS, which is the immediate cause of ER dysfunction. To maximize the therapeutic effect of engineered exosomes, a high‐performance hydrogel with self‐healing, bioadhesive, and exosome‐conjugating properties is applied to encapsulate the engineered exosomes for in vivo application. In vivo, evaluation in diabetic bone defect repair models demonstrates that the engineered exosomes delivering hydrogel system intensively enhance osteogenesis. These findings provide crucial insight into the design and biological mechanism of ER homeostasis‐based tissue‐engineering strategies for diabetic bone regeneration.

## Graphical abstract

Inspired by the discovery that endoplasmic reticulum (ER) dysfunction leads to osteogenic disorder under hyperglycemia, a novel treatment system comprising engineered exosomes and high‐performance hydrogel is utilized to accelerate diabetic bone regeneration. This system integrates with host bone tissues, exhibits excellent self‐healing properties, and provides sustained release of Sep@Exo to support ER homeostasis. This study provides an innovative strategy for diabetic bone defect repair.

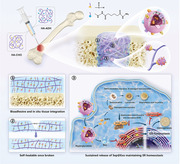

## 1 Introduction

Diabetes mellitus is a chronic metabolic disease characterized by hyperglycemia.^[^ ^1^ ^]^ According to reports from the International Diabetes Federation (IDF), the global prevalence of diabetes is estimated to exceed 10% by 2030.^[^ ^2^ ^]^ Diabetic patients face an elevated risk of bone fracture and delayed bone healing compared to nondiabetic individuals.^[^ ^3^ ^]^ Consequently, there is a substantial need for bone regeneration in these metabolically compromised patients, and it is highly urgent to develop innovative treatment strategies for expedited diabetic bone regeneration.

Previous studies have documented that diabetic hyperglycemia can induce persistent and intense cellular endoplasmic reticulum (ER) stress in multiple tissues, leading to impaired cellular function and subsequent pathophysiological alterations.^[^ ^4^, ^5^, ^6^ ^]^ For bone tissue, existing evidence suggests that mild ER stress is conducive to osteogenesis, while excessive and abnormal ER stress causes osteogenic disorders.^[^ ^7^, ^8^, ^9^, ^10^ ^]^ We speculate that the occurrence of bone loss and impaired bone healing in individuals with diabetes mellitus may be intricately linked to dysfunction in the ER. The ER is a pivotal intracellular membranous organelle that performs multiple essential functions including protein folding, maturation, and trafficking.^[^ ^11^ ^]^ Moreover, the ER interacts with almost all other organelles and contributes greatly to the maintenance of cellular homeostasis.^[^ ^12^ ^]^ Previous studies have demonstrated that the endoplasmic reticulum plays a critical role in bone homeostasis and bone diseases due to its role in the production of collagens and bone growth factors, synthesis of amorphous calcium phosphate, and regulation of calcium signals to induce biomineralization, and so forth.^[^ ^13^, ^14^, ^15^, ^16^ ^]^ Thus, to efficiently repair bone defects in diabetic hyperglycemic microenvironment, ER regulation‐based therapy is expected to be a potential high‐efficiency strategy.

In the past few decades, mesenchymal stem cell (MSC)‐derived exosomes have attracted intense interest and have been widely studied due to their pro‐regenerative properties similar to their parental cells. Recently, researchers found that the therapeutic effect of MSC‐derived exosomes is closely related to the regulation of ER stress. Chen et al. described that MSC‐derived exosomes reduce ER stress to protect beta cells against hypoxia‐induced apoptosis.^[^ ^17^ ^]^ Liao et al. reported that exosomes derived from MSCs downregulate ER stress in nucleus pulposus cells and ameliorate intervertebral disc degeneration.^[^ ^18^ ^]^ Lin et al. supported that MSC‐derived exosomes are conducive to alleviating ER stress and rescuing vertebral endplate chondrocytes.^[^ ^19^ ^]^ However, it is not clear whether MSC‐derived exosomes are able to rescue cellular ER homeostasis in diabetic bone healing. Another important point to consider is that the therapeutic efficacy of pure exosomes is limited, and it is challenging to achieve satisfactory results for diabetic bone regeneration using exosomes alone. Thus, it is imperative to enhance the functionalization of exosomes to upgrade therapeutic efficiency.

Sephin1 (Sep) is a safe and specific small molecule that can give protein folding a helping hand and assist in maintaining ER proteostasis, protecting cells from otherwise lethal ER stress.^[^ ^20^ ^]^ Whereas free drug application usually fails to achieve the desired therapeutic effect due to rapid clearance and limited bioavailability in the local defect areas. To avoid the inefficiency of drug administration, a suitable drug delivery vector is required. Exosomes are natural nanovesicles derived from cells, and they play a crucial role in intercellular communication by facilitating the transport of substances.^[^ ^21^, ^22^ ^]^ Compared with existing synthetic and semisynthetic nano vectors, natural exosomes provide obvious advantages for the efficient delivery of therapeutic agents in terms of biocompatibility, low immunogenicity, high cell internalization efficiency, and so forth.^[^ ^23^, ^24^ ^]^ Thus, incorporating Sep into MSC‐derived exosomes is supposed to be a win‐win strategy.

Considering the inconvenience of multi‐frequency administration in clinical applications, there is an urgent need for a carrier optimized for the sustained release of exosomes to obtain lasting curative effects. Thus, we anticipated a scaffold biomaterial suitable for immobilizing the exosomes to allow their therapeutic effect to be exerted stably and persistently. Hydrogel is a type of hydrophilic, porous, and soft material composed of a 3D polymer network with high water content, mimicking the extracellular matrix commendably. In the past few decades, hydrogels have attracted great attention from researchers due to their wide application prospects in the field of bone tissue engineering.^[^ ^25^ ^]^ Hyaluronic acid (HA) is a natural polysaccharide derived from the extracellular matrix of organisms, which is able to mediate cell signal transduction, promote cell migration, and enhance the regeneration and healing process of injured tissues.^[^ ^26^, ^27^ ^]^ Nevertheless, this type of natural hydrogel is typically fragile and prone to breaking when subjected to external tension. In addition, it easily moves away from the defect region due to the lack of adhesion to tissue, leading to treatment failure. To circumvent all these problems, we synthesized a high‐performance HA‐based hydrogel with self‐healing, bioadhesive, and exosome‐conjugating properties to meet the anticipated requirements of exosome delivery and diabetic bone defect treatment in a clinical setting.

**Scheme 1:**
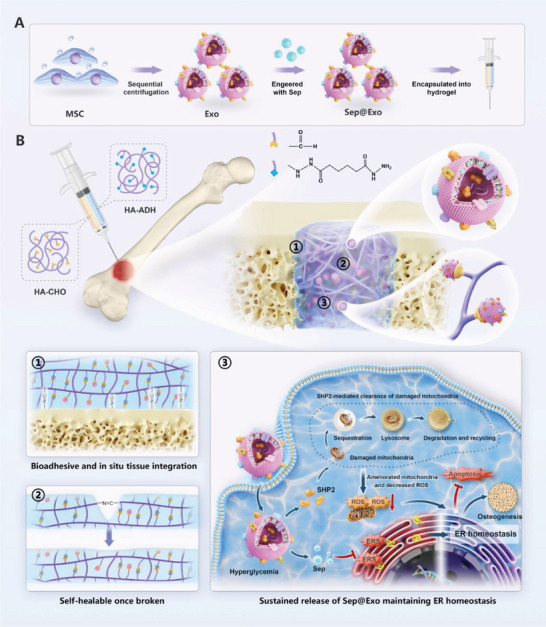
A) Schematic diagram illustrating the fabrication process of the engineered exosomes (Sep@Exo)‐encapsulating hydrogel. B) The Sep@Exo‐encapsulating hydrogel system was injected into femur defects in diabetic rats to facilitate bone regeneration. This system integrates with host bone tissues, exhibits excellent self‐healing properties, and provides sustained release of Sep@Exo to maintain ER homeostasis.

In this study, to achieve successful diabetic bone healing, we constructed a novel engineered exosome loaded with Sep to functionalize MSC‐derived exosomes for modulating ER homeostasis and rescuing impaired MSCs in hyperglycemia. Furthermore, since exosomes were found to exhibit intrinsic therapeutic effects, the potential mechanism was investigated to clarify the detailed events of exosomes on ER homeostasis and osteogenesis. Finally, a self‐healing bioadhesive hydrogel encapsulating engineered exosomes was applied to continuously release the exosomes, modulate ER homeostasis, ameliorate the function of MSCs, and ultimately achieve complete bone regeneration in a well‐established diabetic femoral defect model (**Scheme** **1**).

## 2 Results

### 2.1 Diabetic Osteogenesis Disorder Is Strongly Correlated with ER Dysfunction

**Figure 1:**
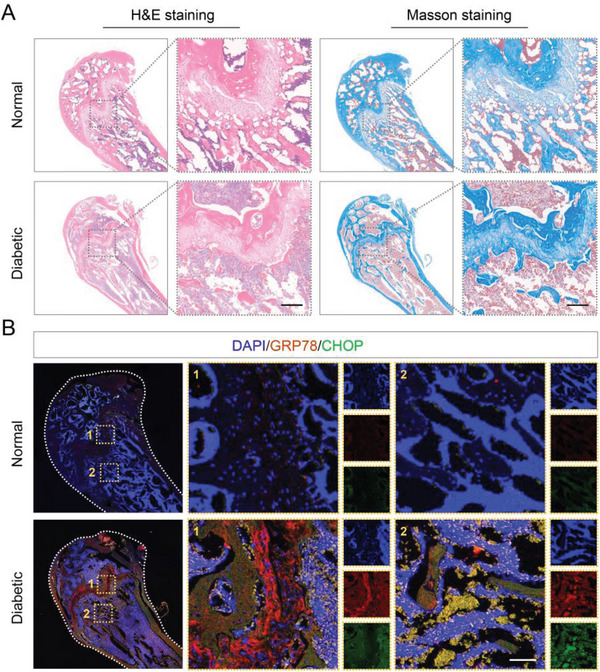
A) H&E staining and Masson trichrome staining of normal and diabetic femurs (*n* = 3). Scale bar: 100 µm. B) Immunofluorescence staining of normal and diabetic femurs (*n* = 3). Scale bar: 50 µm.

To explore the relationship between osteogenic disorder in diabetes mellitus and ER dysfunction, we performed a histological evaluation of diabetic bone tissue. The hematoxylin and eosin (H&E) staining and Masson trichrome staining results showed decreased bone mass and abnormal growth plates in the diabetic group, indicating a bone metabolism disorder (**Figure** **1**A). Then we compared the expression of ER dysfunction‐related proteins in the normal femurs and diabetic femurs through double protein immunofluorescence staining analysis. We found that the expression of GRP78 and CHOP proteins was highly elevated in the femurs from the diabetic group, particularly in and near the femoral growth plate, indicating that the cellular ER state was extremely irregular (Figure 1B). In addition, a high‐glucose (HG) medium with a glucose concentration of 35 mm was used to simulate hyperglycemia in vitro. HG stimulation led to decreased cell viability (Figure S1A, Supporting Information), up‐regulated cell apoptosis levels (Figure S1B, Supporting Information), and compromised osteogenic differentiation (Figure S1C, Supporting Information). Moreover, the expression of ER stress‐related genes was highly increased in the HG group, indicating cellular ER dysfunction (Figure S1D, Supporting Information).

### 2.2 Sep Ameliorates the Function of MSCs by Regulating ER Homeostasis

Sep is a small molecule compound that participates in maintaining the function of the ER by regulating proteostasis, as confirmed by a previous study.^[^ ^20^ ^]^ Here, we aimed to investigate the potential benefits of Sep for MSCs with ER dysfunction in an HG microenvironment. First, we cultured MSCs with varying concentrations of Sep to determine the appropriate dose for cell survival. The results showed that cell viability was unaffected at Sep concentrations of ˂3 µm but decreased significantly when the concentration exceeded 10 µm (Figure S2A, Supporting Information). Therefore, Sep concentrations under 3 µm were selected for subsequent experiments. To investigate the potential of Sep to restore the function of impaired MSCs induced by hyperglycemia, the effects of different concentrations of Sep were assessed using cell proliferation assays, ALP staining, and ALP semiquantitative analysis. The results suggested that the rescue effects of Sep on damaged MSCs were concentration‐dependent, with 1 µm showing the most significant impact (Figure S2B–D, Supporting Information). Therefore, it was hypothesized that a concentration of 1 µm Sep could be optimal for ameliorating the function of impaired MSCs and promoting osteogenic differentiation. Next, we investigated the expression of genes related to ER dysfunction. In comparison to the HG group, these genes were significantly down‐regulated in the Sep‐treated group, implying that Sep is beneficial for maintaining ER homeostasis within the HG microenvironment (Figure S3A, Supporting Information). Previous studies indicated that the increased protein expression of the CHOP is not only a sign of ER dysfunction but also intimately related to apoptosis events.^[^ ^28^, ^29^ ^]^ Here, we observed the co‐localization of the ER dysfunction‐related protein CHOP and the apoptosis‐related marker cleaved caspase‐3 (C‐CAS3) via co‐immunofluorescence staining. The results showed that the protein expression of CHOP and C‐CAS3 exhibited remarkable consistency. Treatment with Sep decreased the expression of both CHOP and C‐CAS3, which demonstrated that Sep could protect MSCs in the HG environment against ER dysfunction‐related apoptosis (Figure S4, Supporting Information). The mRNA expression measurement of osteogenic differentiation‐related genes and OCN immunofluorescence staining further verified our hypothesis (Figure S3B,C, Supporting Information).

### 2.3 Construction and Characterization of Engineered Exosomes and Their Therapeutic Effects on Impaired MSCs

**Figure 2:**
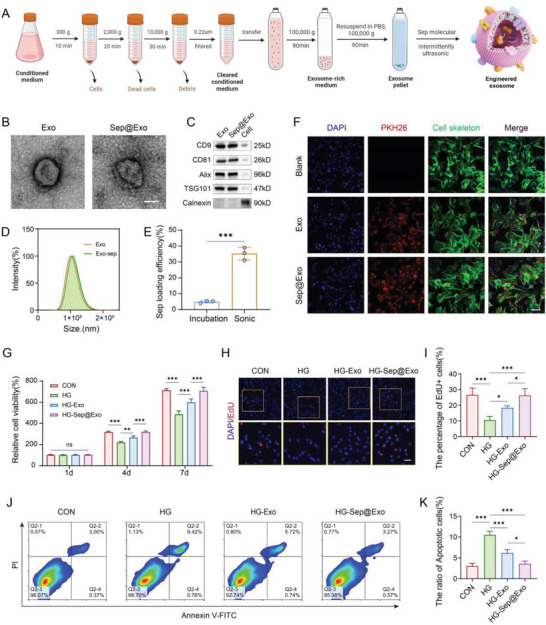
A) Schematic illustration of the preparation process of the engineered exosome (Sep@Exo) (partially created with BioRender.com). B) TEM observation of Exo and Sep@Exo. Scale bar: 50 nm. C) The protein levels of CD9, CD81, Alix, and TSG101 in Exo, Sep@Exo, and MSCs were determined by Western blot. D) The size distribution of Exo and Sep@Exo was determined using dynamic light scattering (DLS). E) Calculation of Sep loading efficiency (*n* = 3). F) Cellular internalization of Exo and Sep@Exo by MSCs (*n* = 3). Scale bar: 100 µm. G) CCK‐8 cell viability of MSCs after treatment with Exo or Sep@Exo in HG medium (*n* = 5). H) and I) EdU staining to show cell proliferation of MSCs after treatment with Exo or Sep@Exo in HG medium (*n* = 4). Scale bar: 50 µm. J, and K) Apoptosis levels of MSCs were measured by flow cytometry after treatment with Exo or Sep@Exo in the HG medium (*n* = 3).

Next, we use Sep to functionalize exosomes derived from MSCs. The MSC‐derived exosomes were extracted and purified according to standard protocol. Then, engineered exosomes loaded with Sep were constructed using intermittent ultrasonic method. **Figure** **2**A illustrates the detailed preparation process of the engineered exosomes. Transmission electron microscopy (TEM) analysis revealed that both the exosome (Exo) and engineered exosome (Sep@Exo) exhibited typical cup‐shaped or spherical morphology (Figure 2B). Western blot analysis confirmed that Exo and Sep@Exo specifically contained CD9, CD81, Alix, and TSG101, but not Calnexin (Figure 2C). The size distribution showed that the Exo and Sep@Exo had similar diameters, ranging from 40–160 nm (Figure 2D). To examine whether the intermittent ultrasound method is superior, we compared the drug loading efficiency of direct incubation and intermittent ultrasound methods. The results demonstrated that the efficiency of the latter was remarkably higher, reaching ≈35%, which was ≈7 times that of the former (Figure 2E). To observe whether Exo and Sep@Exo can be readily taken up by cells, the exosomes labeled with PKH‐26 were incubated with MSCs for 24 h. Immunofluorescence analysis showed that both Exo and Sep@Exo could be internalized effectively by MSCs (Figure 2F). These results indicated that the engineered Sep@Exo was successfully prepared.

Previous studies documented that exosomes can modulate cellular behavior and possess a variety of therapeutic roles.^[^ ^30^, ^31^, ^32^ ^]^ Here, we wanted to investigate whether exosomes have an internal beneficial effect on MSCs in the HG microenvironment. First, we cultured MSCs in an HG medium with varying concentrations of exosomes to determine the appropriate dose for cell survival. The results indicated that the therapeutic effect of exosomes increased as the concentration rose when it was ˂50 µg mL^−1^. However, when the concentration exceeded 50 µg mL^−1^, the therapeutic effect reached saturation and did not continue to increase (Figure S5, Supporting Information). Therefore, for the subsequent experiments, 50 µg mL^−1^ of Exo or 50 µg mL^−1^ of Sep@Exo (containing 1 µm of Sep) was used to rescue the function of impaired MSCs under hyperglycemia. The results showed that the Exo group displayed increased cell viability (Figure 2G) and proliferation rate (Figure 2H,I) compared to the HG group, while the Sep@Exo group showed even better performance. Flow cytometry apoptosis assays exhibited that Exo efficaciously reduced the ratio of apoptotic cells, and the apoptosis rate in the Sep@Exo group almost completely recovered to normal (Figure 2J,K).

### 2.4 Engineered Exosomes Regulate Endoplasmic Reticulum Homeostasis and Rescue Osteogenic Differentiation in Functionally Impaired MSCs

**Figure 3:**
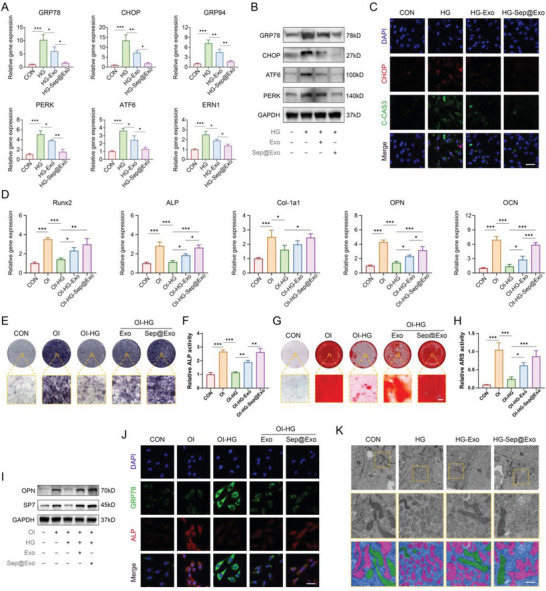
A) Expression of ER dysfunction‐related genes (*n* = 3). B) Expression of ER dysfunction‐related proteins. C) Co‐immunofluorescence staining of C‐CAS3 and CHOP (*n* = 3). Scale bar: 50 µm. D) Expression of osteogenesis‐related genes (*n* = 3). E) ALP staining assay (*n* = 3). Scale bar: 100 µm. F) ALP activity quantitative analysis (*n* = 3). G) ARS staining assay (*n* = 3). Scale bar: 100 µm. H) ARS quantitative analysis (*n* = 3). I) Expression of osteogenesis‐related proteins. J) Co‐immunofluorescence staining of ALP and GRP78 (*n* = 3). Scale bar: 50 µm. K) TEM observation of the subcellular structure (*n* = 3). The red pseudo‐color represents the endoplasmic reticulum, while the green pseudo‐color represents the mitochondria. Scale bar: 500 nm.

Afterward, we examined the state of the ER. The expression of genes and proteins related to ER dysfunction was significantly reduced in the Exo group, while the Sep@Exo group exhibited an even more pronounced effect (**Figure** **3**A,B). Immunofluorescence staining revealed that the protein expression of CHOP and C‐CAS3 decreased synchronously in the exosome‐treated groups, indicating that both Exo and Sep@Exo were beneficial to MSCs in resisting ER stress‐related apoptosis (Figure 3C). When cultured under an osteogenesis‐inducing medium, prominent improvement in ALP activity and mineralized nodule formation (Figure 3E–H) was observed in the Exo group, while Sep@Exo further effectively enhanced the osteogenic function of MSCs. The expression of genes and proteins related to osteogenic differentiation (Figure 3D,I) was consistent with the results mentioned above. In addition, the double staining of GRP78 and ALP directly demonstrated that the osteogenic capability of the impaired MSCs significantly increased with the recovery of ER homeostasis in the exosome‐treated groups (Figure 3J).

To observe the changes in cellular substructures more intuitively, TEM scanning was performed. Under HG conditions, the morphology of the ER appeared highly irregular and fragmented. Additionally, the number of ribosomes attached to its surface decreases, indicating a pathological state and dysfunction. In the Exo group, the severity of ER pathology was alleviated. Surprisingly, the morphology of the ER in the Sep@Exo group was almost similar to that in the control group (Figure 3K). All these results revealed that Sep@Exo had an excellent therapeutic effect on MSCs with ER disorder. Furthermore, the exosomes demonstrate inherent remedial potential for MSCs in the HG microenvironment, beyond the pure role of Sep molecule nano‐carriers.

### 2.5 Exosomes Alleviate ER Stress Through Aiding in the Clearance of Damaged Mitochondria and Decreasing Cellular ROS Production

**Figure 4:**
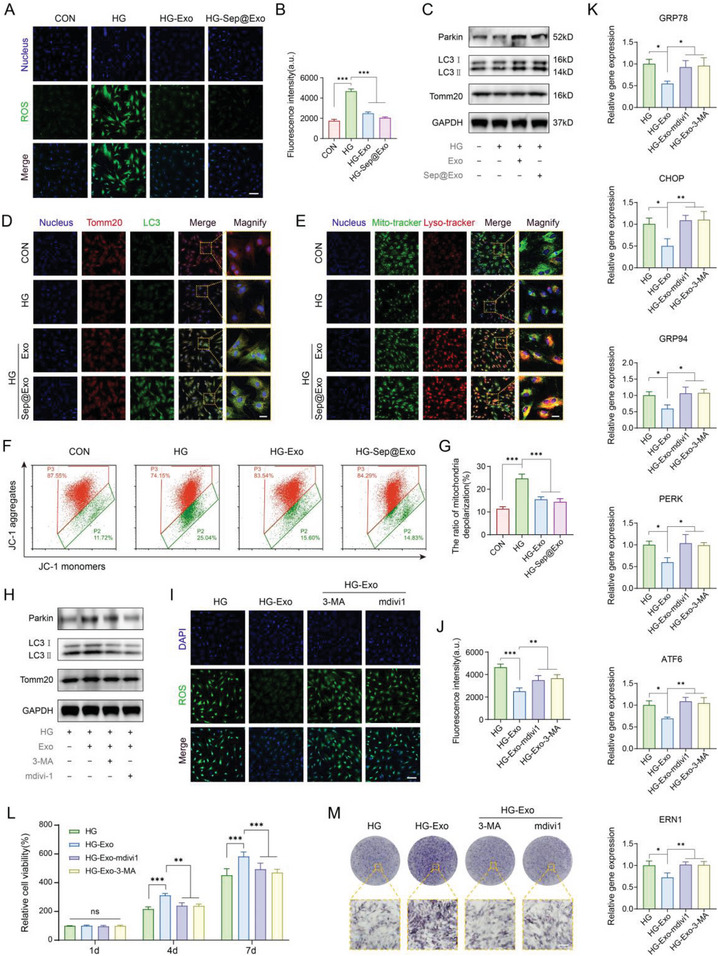
A, B) Intracellular ROS detection (*n* = 5). Scale bar: 100 µm. C) The expression of Parkin, LC3 I/II, and Tomm20 was detected by Western blot. D) The colocalization of mitochondria (stained by Tomm20) and autophagosomes (stained by LC3). Colocalization is represented in yellow (*n* = 3). Scale bar: 25 µm. E) The colocalization of mitochondria (stained with mito‐tracker) and lysosomes (stained with lyso‐tracker). Colocalization is represented in yellow (*n* = 3). Scale bar: 25 µm. F) and G) JC‐1‐mitochondrial membrane potential assay detected by flow cytometry (*n* = 3). H) The expression of Parkin, LC3 I/II, and Tomm20 was detected by Western blot after blocking mitophagy. I) and J) Intracellular ROS detection after blocking mitophagy (*n* = 5). Scale bar: 100 µm. K) ER dysfunction‐related gene expression after blocking mitophagy (*n* = 3). L) CCK‐8 cell viability assay after blocking mitophagy (*n* = 3). M) ALP staining after blocking mitophagy (*n* = 3). Scale bar: 100 µm.

Exosomes are unique and complicated nano‐complexes, and how they assist impaired MSCs in alleviating excessive ER stress and recovering to homeostasis remains unclear. When observing the ultrastructure of cells under an electron microscope, we noticed that besides the transformation of the ER, the morphology of mitochondria also changed obviously. Under the HG microenvironment, the mitochondria were impaired with abnormal morphology and disordered cristae structure but were markedly ameliorated in both the Exo group and the Sep@Exo group. Previous studies have shown that damaged mitochondria serve as the primary source of cellular reactive oxygen species (ROS), while excessive ROS is an essential immediate factor leading to ER dysfunction.^[^ ^33^, ^34^, ^35^ ^]^ Therefore, the fluorescent probe DCFH‐DA was utilized to detect the expression of ROS in each group of cells. We observed a significant increase in ROS production in the HG group. However, it was remarkably down‐regulated in both the Exo group and the Sep@Exo group (**Figure** **4**A,B). We conjecture that the therapeutic mechanism may be closely linked to the enhancement of mitochondrial quality and the decrease in cellular ROS.

Cellular mitophagy refers to the selective removal of damaged mitochondria through autophagy, followed by their decomposition and recycling in lysosomes, which is a vital biological process for maintaining mitochondrial quality control.^[^ ^36^ ^]^ To explore whether exosomes induce mitophagy and modulate mitochondrial homeostasis, we conducted a series of related experiments. It was found that Exo and Sep@Exo elevated the expression of Parkin and promoted the conversion of LC3 I to LC3 II in MSCs (Figure 4C), suggesting an enhanced process of mitophagy. The expression of the mitochondrial marker Tomm20 was also augmented, indicating the enhancement in mitochondrial regeneration. Immunofluorescence staining visually showed that the fusion of mitochondria with autophagosomes and lysosomes was strengthened in the Exo and Sep@Exo treated groups (Figure 4D,E). Damaged mitochondria are usually accompanied by a decline in mitochondrial membrane potential (MMP). JC‐1 analysis showed that Exo and Sep@Exo effectively mitigated the decrease in MMP caused by the HG microenvironment in MSCs (Figure 4F,G). All of these results suggest that exosomes are critical for the activation of mitophagy, which is not significantly affected by Sep loading.

To clarify the involvement of activated mitophagy in exosome‐mediated ER protection, we used mitophagy inhibitors mdivi‐1 and 3‐methyladenine (3‐MA) to block mitophagy. Western blot experiments confirmed the inhibitory effect of 3‐MA and mdivi‐1 on mitophagy (Figure 4H). As shown in the results, the MMP failed to recover, and the exosome‐induced reduction of cellular ROS significantly rebounded after blocking mitophagy (Figure 4I,J; Figure S6, Supporting Information), accompanied by consequent ER dysfunction (Figure 4K). In addition, the cell activity and osteogenic differentiation up‐regulated by exosomes decreased (Figure 4L,M; Figure S7, Supporting Information). These results supported our hypothesis that exosomes alleviate ERS through activation of mitophagy and decreasing cellular ROS production.

### 2.6 Exosomes Exert Therapeutic Effects Through Directly Delivering the SHP2 Protein

**Figure 5:**
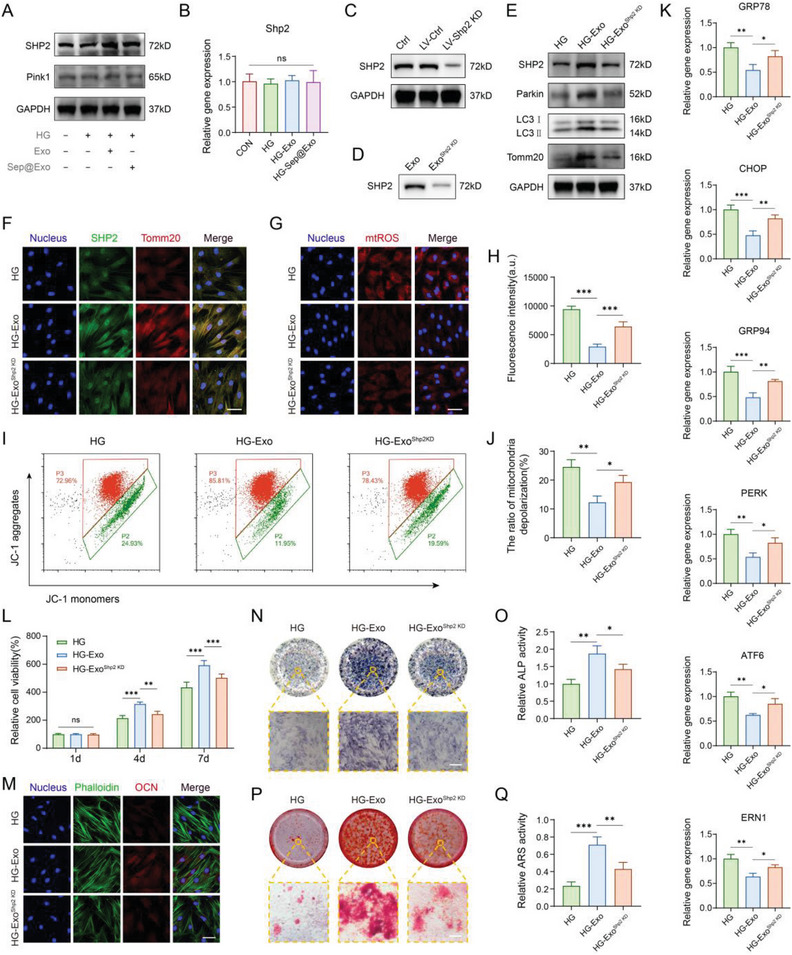
A) The expression of Pink1 and SHP2 detected by Western blot. B) The mRNA level of Shp2 detected by RT‐PCR (*n* = 3). C) Western blot showed that LV‐Shp2 KD effectively inhibited SHP2 expression in MSCs. D) The level of SHP2 in exosomes derived from LV‐Shp2 KD‐treated MSCs was markedly reduced. E) The expression of SHP2, Parkin, LC3 I/II, and Tomm20 was detected by Western blot. F) Co‐immunofluorescence staining of SHP2 and Tomm20 (*n* = 3). Scale bar: 50 µm. G) and H) MitoSOX staining for observing mitochondrial ROS (*n* = 5). Scale bar: 50 µm. I) and J) JC‐1‐mitochondrial membrane potential assay detected by flow cytometry (*n* = 3). K) Expression of ER dysfunction‐related genes (*n* = 3). L) CCK‐8 cell viability assay (*n* = 3). M) Immuno‐fluorescence staining of OCN. Scale bar: 50 µm. N) ALP staining assay (*n* = 3). Scale bar: 100 µm. O) ALP activity analysis (*n* = 3). P) ARS staining assay (*n* = 3). Scale bar: 100 µm. Q) ARS quantitative analysis (*n* = 3).

Next, we attempted to determine how exosomes activate mitophagy and aid in the clearance of damaged mitochondria in MSCs. Pink1 and Parkin are classic partners that enhance the mitophagy process. However, we were surprised to find that although exosomes promoted the expression of Parkin, Pink1 did not show a significant increase. Previous studies reported that SHP2 is another protein that interacts with parkin to positively regulate mitophagy.^[^ ^37^ ^]^ The results of the Western blot assay showed obvious upregulation of SHP2 protein levels in the Exo and Sep@Exo groups (**Figure** **5**A). However, real‐time PCR analysis showed that the mRNA expression of SHP2 did not change significantly (Figure 5B), indicating that exosomes may directly provide protein SHP2 to elevate its level in recipient cells without genetic regulation.

To verify that SHP2 in exosomes contributes to exosome‐induced mitophagy activation, ROS reduction, ER stress mitigation, and rescue of impaired MSCs, we examined whether the blockage of SHP2 delivery could hinder the exosome‐induced therapeutic effect. A lentivirus vector incorporating short hairpin RNA (shRNA) targeting Shp2 was constructed and utilized to knock down Shp2 expression in MSCs. The analysis of the sequencing results demonstrated the successful construction of the Rat_Shp2‐shRNA interference vector (Figure S8, Supporting Information). The lentivirus transfection efficiency was evaluated with fluorescence microscopy and further detected by flow cytometry (Figure S9A,B, Supporting Information). SHP2 expression was significantly decreased, as demonstrated by PCR and Western blot analysis (Figure 5C; Figure S9C, Supporting Information). Concomitantly, exosomes generated by MSCs^Shp2 KD^ (Exo^Shp2 KD^) contained significantly reduced levels of SHP2 (Figure 5D). Exo^Shp2 KD^ treatment failed to upregulate SHP2 or mitophagy‐related proteins in impaired MSCs. Moreover, the expression of Tomm20 was not significantly increased in the Exo^Shp2 KD^ group (Figure 5E). The results of immunofluorescence staining of SHP2 and Tomm20 were consistent with the above description (Figure 5F). MitoSOX Red is a highly selective fluorescent molecular probe targeting the superoxide production of mitochondria in living cells. We detected the changes in mitochondrial ROS (mtROS) of each group using MitoSOX Red. The Exo group demonstrated a strong scavenging ability for accumulated mtROS, while the Exo^Shp2 KD^ group showed a compromised reduction of mtROS compared to the Exo group (Figure 5G,H). Moreover, the MMP in the Exo^Shp2 KD^ group showed compromised recuperation (Figure 5I,J). We speculate that this may be the result of damaged mitochondria not being effectively removed in the Exo^Shp2 KD^ group. Furthermore, Exo^Shp2 KD^ showed a declined capability to alleviate ER stress compared to the Exo group (Figure 5K; Figure S10, Supporting Information). In addition, impaired cell viability of MSCs failed to be rescued in the Exo^Shp2 KD^ group (Figure 5L). The capacity for osteogenic differentiation was attenuated in the Exo^Shp2 KD^ group, as assessed by OCN immunostaining (Figure 5M), ALP activity (Figure 5N,O), and mineralized nodule formation (Figure 5P,Q). These data verify our hypothesis that exosomes directly deliver the protein SHP2 to MSCs for mitophagy activation and mtROS scavenging, contributing to the maintenance of ER homeostasis.

In brief, on the one hand, Sep molecules loaded by engineered exosomes contribute to maintaining ER homeostasis by directly working on reducing the ER burden and mitigating ER stress as reported previously; on the other hand, SHP2 protein inherent in exosomes can indirectly modulate ER homeostasis by activating mitophagy to eliminate mtROS and diminish the inducement of ER disorder. Both sides coordinate and cooperate in harmony, perfectly maintaining the ER homeostasis and cellular function of MSCs.

### 2.7 Synthesis and Characterization of the Hydrogel

To magnify the therapeutic effects of engineered exosomes, we anticipated a hydrogel with high performance to encapsulate them for in vivo application. The ideal candidate hydrogels are expected to possess bioadhesive properties and excellent tissue integration, protecting the hydrogels from detaching from the defect site. They should also have appropriate mechanical strength and stability, adapt to the complexity of the local defect, and be able to self‐heal to maintain their integrity once broken. Finally, they should be capable of well‐enclosing exosomes to achieve sustained and controlled release.

**Figure 6:**
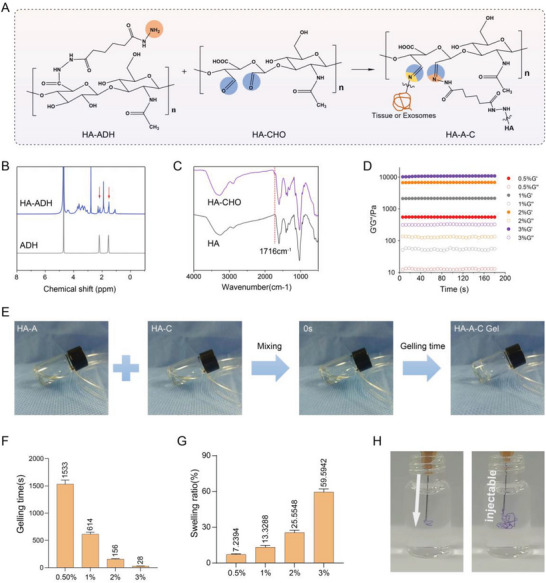
A) Schematic diagram of the reaction strategies for hydrogel gelation, exosome conjugation, and tissue integration. B) ^1^H NMR spectra of HA‐ADH and ADH. C) FTIR spectra of HA‐CHO and HA. D) Storage modulus and loss modulus of HA‐A‐C hydrogels with different solid contents (*n* = 3). E) Schematic illustration of Gelling time detection. F) Gelling time of the HA‐A‐C hydrogels with different solid contents (*n* = 3). G) Swelling ratio of the HA‐A‐C hydrogels with varying solid contents in PBS buffer (*n* = 3). H) The HA‐A‐C hydrogels were injected into PBS to observe injectability.

HA is a natural polymer that is ubiquitously expressed in the extracellular matrix and has already been employed in tissue engineering to accelerate bone regeneration. Nevertheless, this kind of natural hydrogel is usually fragile and easily breaks when subjected to external tension. Here, we synthesized a high‐performance HA‐A‐C hydrogel based on HA, which consists of dual components including ADH‐grafted HA‐ADH and aldehyde‐modified HA‐CHO. These polysaccharide derivatives can undergo self‐assembled crosslinking through ADH‐CHO interactions in a mild and rapid reaction, forming dynamic and reversible C═N covalent bonds to achieve self‐healing properties. The aldehydes are able to conjugate with amine groups on exosomes and employ imine anchoring to connect to host tissues. The reaction principle of the HA‐A‐C hydrogel is described in **Figure** **6**A. HA‐ADH was chemically confirmed using nuclear magnetic resonance (NMR), which revealed new resonances corresponding to the protons on ADH in the ^1^H NMR spectra (Figure 6B). Successful synthesis of HA‐CHO was evidenced by Fourier transform infrared spectroscopy (FTIR) analysis. It appeared a new peak at ≈1716 cm^−1^ in the FTIR spectrum of HA‐CHO compared to HA; this signal is a characteristic of the C═O stretching of the aldehyde group in HA‐CHO (Figure 6C).

Next, we explored the properties of HA‐A‐C hydrogel with different solid concentrations. The rheological time sweep tests revealed that the storage modulus of the hydrogel augmented with the increase of solid content, 3 wt.% of which can exceed 10 000 Pa, indicating excellent mechanical performance and stability of the hydrogel (Figure 6D). The gelling time of HA‐A‐C hydrogel was observed at varying concentrations in order to find an optimized one for preparation. The concentrations varied from 0.5 to 3 wt.%, and the gelling time of the HA‐A‐C hydrogel decreased from over 25 min to under 30 s (Figure 6E,F). Nevertheless, the hydrogel with a higher solid content exhibited a larger swelling ratio, ranging from ≈7% to 60% (Figure 6G). Considering the balance of the mechanical performance, proper gelling time, and swelling behavior, 2 wt.% HA‐A‐C hydrogel was chosen for the subsequent experiments (unless otherwise specified). The excellent injectability of the HA‐A‐C hydrogel was demonstrated by injecting it into a bottle containing PBS solution (Figure 6H). Moreover, we detected the hydrogel degradation behavior according to a previous study,^[^ ^38^ ^]^ the HA‐A‐C hydrogel exhibited proper biodegradability when exposed to hyaluronidase (Figure S11, Supporting Information).

### 2.8 High Performance and Biocompatibility the Hydrogel

**Figure 7:**
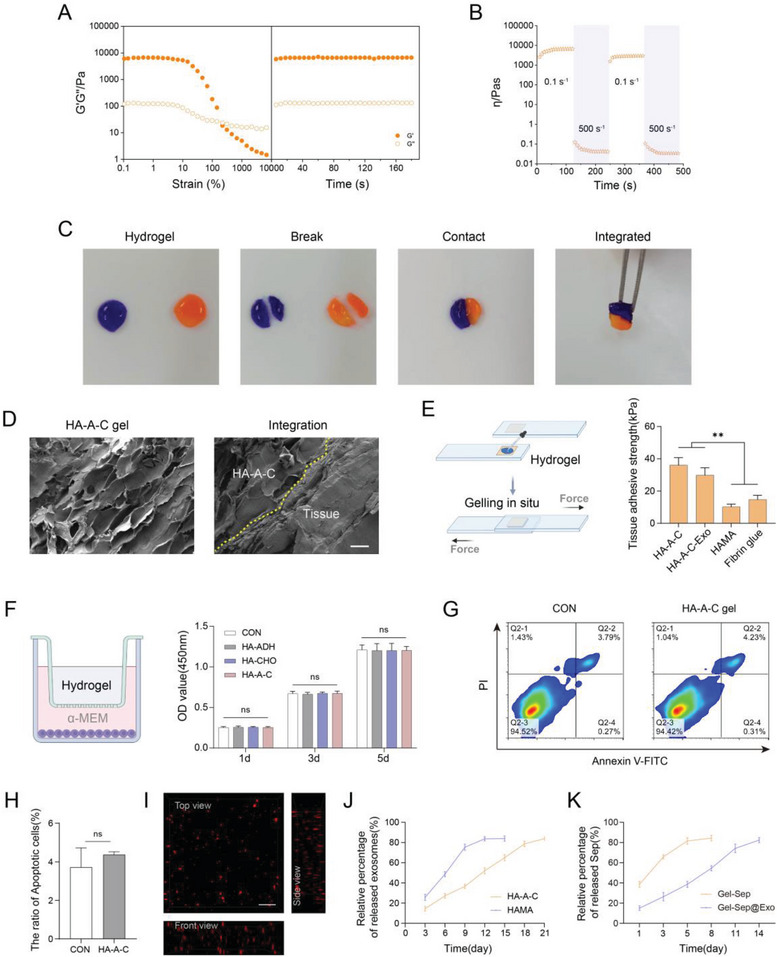
A) Oscillation strain sweep followed by time sweep revealing the self‐healing property of HA‐A‐C hydrogels (*n* = 3). B) Variation in viscosity of HA‐A‐C hydrogel under alternate shear forces of 0.1 s^−1^ and 500 s^−1^ (*n* = 3). C) Integration of two broken hydrogel tablets after mutual interaction. D) SEM observation of the integration of the HA‐A‐C hydrogel with tissue (yellow dashed line indicates the boundary) (*n* = 3). Scale bar: 50 µm. E) Adhesive strength of different hydrogels to tissue detected by lap shear tests (*n* = 3). F) Cell viability of MSCs incubated with the gel precursor solution and the formed hydrogel in the transwell model (*n* = 3). G) and H) Cell apoptosis rate of MSCs seeded on the HA‐A‐C hydrogel surface measured by flow cytometry (*n* = 3). I) Exosome distribution in the HA‐A‐C hydrogel (*n* = 3). Scale bar: 1 µm. J) Exosome release profile of the HA‐A‐C hydrogel (*n* = 3). K) Sep release profile of the HA‐A‐C hydrogel (*n* = 3).

To examine the self‐healing effect of the HA‐A‐C hydrogel, strain sweep oscillatory tests ranging from 1% to 10000% were performed on it, and the rupture of the hydrogel structural network was evidenced by higher G’’ compared with G’ upon increasing strain. After allowing the destroyed hydrogel to heal for 15 min, a time sweep oscillatory test revealed that the broken hydrogel was recovered to initial G’/G’’, demonstrating remarkable self‐healing performance (**Figure** **7**A). The rapid variation in viscosity of the HA‐A‐C hydrogel under alternate step shear rates of 0.1 and 500 s^−1^ also illustrated that the hydrogel possesses excellent shear thinning and structural recovery performance (Figure 7B). Furthermore, for macroscopic observation of self‐healing capacity, the HA‐A‐C hydrogels gelling in tablet shape and dyed with different colors were cut into two parts and tried to reconnect after exchanges. After self‐healing for 3 min, the reassembled pieces exhibited an ambiguous boundary at the healed interface and formed a new intact tablet shape (Figure 7C).

To evaluate the superior tissue integration capability of the HA‐A‐C hydrogel, scanning electron microscopy (SEM) analysis was used to characterize hydrogel adhesion to muscle tissue. The firm adhesion between the hydrogel and the tissue was confirmed by their seamless contact (Figure 7D). Additionally, standard lap‐shear measurements were conducted to determine the tissue adhesive strength of the HA‐A‐C hydrogel. The results showed that the HA‐A‐C hydrogel performed higher adhesive strength (≈35 kPa) than the 2% HAMA hydrogel (≈10 kPa) and commercial fibrin glue (≈15 kPa). After the hydrogel was loaded with 50 µg mL^−1^ exosomes, the adhesive strength decreased slightly, which might be correlated with the occupation of some aldehyde group sites by the exosomes. Nevertheless, it was still stronger than 2% HAMA and commercial fibrin glue (Figure 7E).

To evaluate the cytocompatibility of the HA‐A‐C hydrogel, MSCs were incubated with the HA‐A‐C precursors and the formed hydrogel using a transwell model. The results showed that neither precursors nor the formed hydrogel exerted adverse effects on cell viability (Figure 7F). Furthermore, flow cytometry analysis revealed no significant variations in the cell apoptosis rate after 3 days of direct cultivation on the formed HA‐A‐C hydrogel surface (Figure 7G,H).

The results of 3D confocal fluorescence microscopy imaging exhibited that the exosomes labeled with red fluorescence were distributed homogeneously in the HA‐A‐C hydrogel, demonstrating the excellent exosome‐carrying capability of the hydrogel (Figure 7I). Then, we investigated the exosome release profile of the HA‐A‐C hydrogel. Compared to HAMA, the exosomes encapsulated in the HA‐A‐C hydrogel were released in a longer sustained manner, which might be attributed to the conjugation of exosomes with aldehyde groups (Figure 7J). Compared with free Sep loading into HA‐A‐C hydrogel, the release profile of Sep in Sep@Exo hydrogel system appeared to have a longer duration (Figure 7K). All these data demonstrated that the HA‐A‐C hydrogel is a potentially remarkable assistant for amplifying the therapeutic effect of the engineered exosomes.

### 2.9 High‐Performance Hydrogel‐Encapsulated Engineered Exosomes Enhances Diabetic Bone Regeneration In Vivo

**Figure 8:**
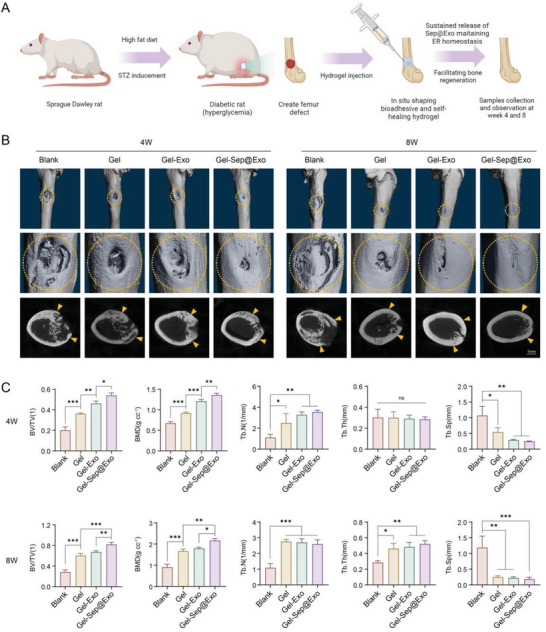
A) Schematic illustration showing the construction of diabetic rats and the expected effect of Gel‐Sep@Exo in the rat diabetic hyperglycemic microenvironment (created with BioRender.com). B) Micro CT evaluation of bone regeneration using femur bone defects implanted with different hydrogels in the diabetic rat model (*n* = 3). C) Quantitative analysis of newly formed bone in the studied groups (*n* = 3).

Ultimately, in order to evaluate the therapeutic potential of the engineered exosome‐encapsulating hydrogel system in vivo, we established a type 2 diabetic rat model via a high‐fat diet and STZ inducement. Then different groups of hydrogels were injected into rat diabetic femoral defects, with the blank group serving as the control. After implantation for 4 and 8 weeks, the samples were collected for micro‐CT scanning and subsequent histological analysis (**Figure** **8**A). As the 3D micro‐CT reconstructed images and 2D sectional images showed, the amount of new bone formation in the Blank, Gel, Gel‐Exo, and Gel‐Sep@Exo groups showed a successively increasing tendency at both weeks 4 and 8. The Gel‐Sep@Exo group witnessed a large area of new bone filling in the defects, exhibiting the best bone regeneration performance among all groups, while only a small amount of new bone was observed in the blank group indicating delayed bone healing (Figure 8B). In micro‐CT quantitative analysis, the Gel‐Sep@Exo group exhibited the most regenerated bone mass compared to the other groups at both weeks 4 and 8, with highest bone volume/tissue volume ratio (BV/TV), bone mineral density (BMD), trabecular number (Tb. N), trabecular thickness (Tb. Th), and lowest trabecular separation (Tb. Sp) (Figure 8C).

**Figure 9:**
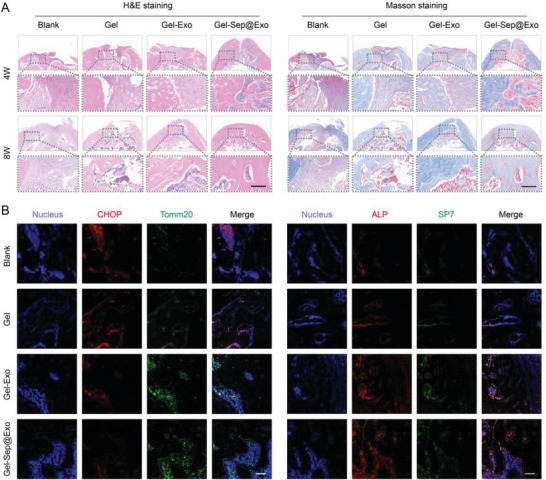
A) H&E and Masson's trichrome staining of the decalcified defect region (*n* = 3). Scale bar: 200 µm. B) Immunofluorescence staining of CHOP and Tomm20 was conducted to observe ER homeostasis and mitochondrial status in the bone defect region. Immunofluorescence staining of ALP and SP7 was performed to visualize new bone regeneration in the bone defect region (*n* = 3). Scale bar: 100 µm.

H&E staining and Masson's trichrome staining supplied additional complementary details concerning the regenerated bone tissues. As shown in the results, fibrous tissues without new bone formation appeared in most of the defect areas in the blank group at both weeks 4 and 8. The Gel group exhibited slightly more bone formation than the blank control group. The Gel‐Exo group performed better than the Gel group. Notably, the bone tissue in the Gel‐Sep@Exo group was almost completely regenerated at week 8, as evidenced by the large consecutive bone regions that were not present in the other groups (**Figure** **9**A). Furthermore, the state of organelles in the different groups was examined by immunofluorescence staining of CHOP and Tomm20. The results showed that the Gel‐Sep@Exo group exhibited the lowest level of CHOP and the highest level of Tomm20, indicating remarkable improvement of mitochondria and effective maintenance of ER homeostasis. Moreover, immunofluorescence staining of the osteogenesis markers ALP and SP7 further demonstrated extensively accelerated bone regeneration in the Gel‐Sep@Exo group (Figure 9B).

The in vivo biosafety of the Gel‐Sep@Exo was assessed by observing major metabolic organs (including lung, heart, kidney, liver, and spleen) of all the studied groups. As shown in the histological images, no pathological abnormalities were observed in any of the detected metabolic organs, indicating excellent in vivo biocompatibility of the hydrogel and Sep@Exo (Figure S12, Supporting Information).

## 3 Discussion

Evidence from both laboratory and clinical settings has substantiated that diabetic hyperglycemia impairs bone formation resulting from cellular dysfunction and decreased bone turnover.^[^ ^3^ ^]^ The unfavorable microenvironment directly influences clinical bone defect treatment and leads to delayed bone healing. As the largest organelle in the cell, the ER possesses complicated and diverse functions and plays a pivotal role in numerous critical cellular physiological events, while ER disorder triggers cellular functional disturbance.^[^ ^11^, ^39^, ^40^ ^]^ An ER‐based therapy that can regulate overload stress and functional alterations arising from hyperglycemia and establish an optimum intracellular microenvironment for osteogenesis may offer remarkable prospects in developing new strategies for stimulating bone regeneration.

In this study, we incorporated Sep into exosomes derived from MSCs to fabricate novel engineered exosomes to modulate ER homeostasis and restore the cellular function of MSCs in the HG microenvironment. Exosomes derived from MSCs have attracted increasing attention due to their appealing therapeutic potential in bone tissue regeneration.^[^ ^41^, ^42^, ^43^, ^44^, ^45^ ^]^ Here, we found that normal MSC‐derived exosomes were capable of mitigating the ER stress of MSCs suffering from hyperglycemia. Additionally, we attempted to unveil the mystery of the exact mechanism of exosomes. The ER and mitochondria are two vital fundamental cellular organelles that interact closely and frequently, collaboratively regulating many essential cellular processes such as ion and lipid transfer, signal transduction, and membrane dynamics.^[^ ^46^ ^]^ Mitochondrial disruption easily leads to ER stress activation, since damaged mitochondria would release overwhelming ROS from a leaky electron transport chain (ETC).^[^ ^47^ ^]^ It has been well documented that an HG environment in diabetes mellitus would cause mitochondrial damage and the production of excessive cellular ROS.^[^ ^48^, ^49^ ^]^ The obvious alterations of mitochondria observed under TEM and cellular ROS detection also confirmed it. Interestingly, we discovered that MSC‐derived exosomes greatly contributed to mitochondrial quality improvement and cellular ROS elimination.

Mitophagy is a crucial process for mitochondrial quality control, which selectively scavenges impaired mitochondria through a specialized form of autophagy.^[^ ^50^, ^51^ ^]^ We investigated the potential role of mitophagy on mitochondrial quality improvement and ER stress alleviation in exosome‐treated MSCs cultivated in HG conditions. The results illustrated that MSC‐derived exosomes significantly induced mitophagy of the impaired MSCs, along with down‐regulation of cellular ROS and recovered MMP. Nevertheless, consequent ROS reduction, ER stress mitigation, and functional therapeutic effects were not achieved when mitophagy was inhibited. Furthermore, we discovered that MSC‐derived exosomes were able to maintain ER homeostasis and rescue impaired MSCs via directly delivering SHP2 to activate the mitophagy pathway and eliminate mtROS. It is a previously unknown mechanism of MSC‐derived exosomes in facilitating the function of impaired MSCs and diabetic bone regeneration. We conclude that the engineered exosomes improve ER homeostasis and mitochondrial quality largely in parallel with ameliorating the cellular function of MSCs that have the potential to differentiate into osteoblasts under diabetic hyperglycemia. These findings contribute to a deeper understanding of the cellular damage mechanisms in MSCs in diabetes and provide new insights for the development of related therapeutic strategies.

Injectable hydrogels, valued as biomimetic biomaterials due to their remarkable similarity to the extracellular matrix, offer distinctive advantages for localized minimally invasive applications. Here, we have fabricated a multifunctional hydrogel to encapsulate engineered exosomes for in vivo application. The dual‐component hydrogel can perform three key functions: self‐healing, bio‐adhesion, and exosome conjugation.

As an advanced property of hydrogels, the self‐healing capability can offer various profits for a tissue regenerative material. One of the values lies in injectable and flexible delivery process which can achieve minimally invasive treatment. The fit‐to‐shape effect of self‐healing hydrogel facilitates adaptation to complex bone defect shapes, especially the bone defects in the oral and maxillofacial regions. Moreover, the self‐healing hydrogel is capable of maintaining its integrity spontaneously once it is destroyed by external force.

Another pragmatic function is bio‐adhesiveness since the retention and integration of the implanted bioactive material at the defect site is a critical factor for the success of bone tissue regeneration. The aldehyde groups in the fabricated hydrogel are able to covalently anchor to the amino groups that exist on the surface of the bone defect, forming tissue adhesion and integration along with the gelling process of the HA‐A‐C hydrogel without any additional operation.

Aldehyde groups can also be used as conjugation sites for amino groups on the surface of exosomes, enabling controlled and sustained release of exosomes, which is of great significance in achieving long‐term therapeutic effects.

The experiments conducted in diabetic bone defect models strongly support the conclusion that our Gel‐Sep@Exo therapeutic system could facilitate bone tissue repair and shorten bone healing time.

## 4 Conclusion

In conclusion, we developed a novel therapeutic platform aimed at accelerating diabetic bone regeneration based on ER homeostasis modulation. Diabetic bone defects continuously suffer from hyperglycemia and ER stress, which suppresses osteogenesis. To counteract unfavorable diabetic bone healing conditions, the small molecule Sep was engineered into MSC‐derived exosomes. Sep could alleviate ER stress by maintaining ER proteostasis. MSC‐derived exosomes can not only serve as a delivery vector for Sep, but also provide protein SHP2 to recipient cells to activate mitophagy and scavenge overproduced mtROS, so as to maintain cellular ER homeostasis in cooperation with Sep. For in vivo application, we successfully designed and constructed a diabetic bone defect repair treatment system comprising engineered exosomes and high‐performance hydrogel. This hydrogel system is equipped with multiple functions anticipated in a clinical setting. It exhibits convenient injectability and in‐situ gelling capability. It employs imine anchoring to connect to host tissues to obtain excellent tissue integration. The dynamic reversible crosslinking bond endows it with excellent self‐healing performance. Last but not least, the exosome‐conjugating property renders exosome release in a controlled manner and exerts enduring therapeutic effects for maintaining ER homeostasis and expediting osteogenesis. Thus, our study demonstrated that the user‐friendly therapeutic system consisting of engineered exosomes and high‐performance hydrogel holds potential for clinical application and may further inspire the development of innovative treatments for diabetic bone diseases. Moreover, the versatility of this strategy goes beyond diabetic bone regeneration and provides promise for tissue regeneration in other related contexts.

## Conflict of Interest

The authors declare no conflict of interest.

## Supporting information

Supporting Information

## Data Availability

The data that support the findings of this study are available from the corresponding author upon reasonable request.
