# Supplementary material for: High‐Performance Hydrogel‐Encapsulated Engineered Exosomes for Supporting Endoplasmic Reticulum Homeostasis and Boosting Diabetic Bone Regeneration
Source: Adv Sci (Weinh). 2024 Feb 21;11(17):2309491. doi: 10.1002/advs.202309491 (PMC11077675; doi:10.1002/advs.202309491)
Supplement: Supplementary file 1 — Supporting Information [file ADVS-11-2309491-s001.pdf]

ADVANCED SCIENCE
Open Access

Supporting Information

for *Adv. Sci.*, DOI 10.1002/advs.202309491

High-Performance Hydrogel-Encapsulated Engineered Exosomes for Supporting Endoplasmic Reticulum Homeostasis and Boosting Diabetic Bone Regeneration

Yulan Liu, Sihan Lin, Zeqian Xu, Yuqiong Wu, Guifang Wang, Guangzheng Yang, Lingyan Cao, Haishuang Chang, Mingliang Zhou and Xinquan Jiang**

Supporting Information

High-Performance Hydrogel-Encapsulated Engineered Exosomes for Supporting Endoplasmic Reticulum Homeostasis and Boosting Diabetic Bone Regeneration

Yulan Liu[#], Sihan Lin[#], Zeqian Xu, Yuqiong Wu, Guifang Wang, Guangzheng Yang, Lingyan Cao, Haishuang Chang, Mingliang Zhou[*], Xinquan Jiang[*]

Y. Liu, S. Lin, Z. Xu, Y. Wu, G. Wang, G. Yang, L. Cao, M. Zhou, X. Jiang
Department of Prosthodontics, Shanghai Ninth People's Hospital, Shanghai Jiao Tong University School of Medicine; College of Stomatology, Shanghai Jiao Tong University; National Center for Stomatology; National Clinical Research Center for Oral Diseases; Shanghai Key Laboratory of Stomatology; Shanghai Research Institute of Stomatology; Shanghai Engineering Research Center of Advanced Dental Technology and Materials, Shanghai 200125, China
Email: mingliangzhou@aliyun.com; xinquanjiang@aliyun.com
H. Chang
Shanghai Institute of Precision Medicine, Shanghai Ninth People’s Hospital, Shanghai Jiaotong University School of Medicine, Shanghai 200125, China

Materials and Methods

Materials

All chemical reagents were purchased from commercial retailers and used without further purification, unless otherwise specified. Hyaluronic acid (HA), $NaIO_4$, Adipic acid dihydrazide (ADH), 1-(3-Dimethylaminopropyl)-3-ethylcarbodiimide Hydrochloride (EDC), and 1-Hydroxybenzotriazole Monohydrate (HOBT) were purchased from Macklin (Shanghai, China). Streptozotocin (STZ), D-glucose, and

Sephin1 were bought from Sigma-Aldrich (St. Louis, USA). Alpha Minimum Essential Medium (MEM), trypsin, penicillin-streptomycin, and fetal bovine serum (FBS) were obtained from Gibco (Grand Island, USA).

Table S1 Antibodies used in this study:

Antibodies	Company	Cat:	Dilution
GRP78	Abcam	ab21685	IHC(1:200), ICC(1:200), WB(1:1000)
CHOP	CST	#2895	IHC(1:100), ICC(1:200), WB(1:1000)
C-Cas3	CST	#9664	ICC(1:400)
CD9	CST	#98327	WB(1:1000)
CD81	Abcam	ab109201	WB(1:1000)
Alix	Abcam	ab235377	WB(1:1000)
TSG101	Abcam	ab125011	WB(1:1000)
Calnexin	Abcam	ab133615	WB(1:1000)
OCN	R&D systems	MAB1419	ICC(1:100)
ALP	R&D systems	AF2910	ICC(1:100)
ATF6	Proteintech	66563-1-Ig	WB(1:1000)
PERK	CST	#3192	WB(1:1000)
SP7	Abcam	ab209484	IHC(1:200), WB(1:1000)
OPN	Proteintech	22952-1-AP	WB(1:1000)
Tomm20	Abcam	ab283317	IHC(1:500), ICC(1:500), WB(1:1000)
LC3	CST	#12741	ICC(1:200), WB(1:1000)
Parkin	Abcam	ab77924	WB(1:1000)
Pink1	Proteintech	23274-1-AP	WB(1:500)
SHP2	Abcam	ab300579	ICC(1:50), WB(1:1000)
GAPDH	CST	#5174	WB(1:1000)

Animals

C57BL/6 mice and Sprague-Dawley (SD) rats were acquired from the Animal Center of Shanghai Ninth People's Hospital. The in vivo experiments carried out in this research were approved by the Animal Experimental Ethical Committee of Ninth People's Hospital, Shanghai Jiao Tong University (SH9H-2019-A167-1).

Diabetic mice model

Three-week-old C57BL/6 mice were fed with a high-fat diet for 4 weeks and then injected with STZ dissolved in sodium citrate buffer intraperitoneally for five days at a dose of 45 mg/kg/day as previously reported.[1] After one week, the blood glucose levels were detected and mice consistently exceeding 16.7mM for one week were considered successful models for diabetes. The normal group consisted of age-matched C57BL/6 mice that were fed a regular diet and injected with a buffer solution.

Histological analysis and immunofluorescence staining

Specimens were collected and fixed with formalin. After decalcification, the specimens were embedded in paraffin and sectioned for histological evaluation. H&E staining (Servicebio, China) and Masson trichrome staining (Servicebio, China) were performed in accordance with the manufacturer's instructions. For immunofluorescence staining, the slices were dewaxed and hydrated using standard procedures, followed by antigen retrieval in a sodium citrate buffer at 98 °C for 5 min. Next, the slices were blocked for 1 h and co-incubated with specific primary antibodies at 4 °C overnight, followed by co-incubation with secondary antibodies for 1 h. DAPI staining was then performed for 10 min at room temperature. Finally, the slices were captured using a confocal laser scanning microscope (Leica, Germany). Antibodies used in this study are listed in Table S1.

Cell isolation and culture

The femurs and tibias of 3-week-old male Sprague Dawley (SD) rats were used to isolate bone marrow MSCs as previously described.[2] Briefly, after removing both ends

of the femurs and tibias, cells were washed from the bone marrow and cultured in a normal-glucose medium (α-MEM with 5.5 mM D-glucose) containing 10% FBS and 1% penicillin-streptomycin at 37 °C in a humidified incubator with 5% CO2. The MSCs at passages 2-4 and with 80-90% confluency were used for the subsequent experiments. In order to mimic hyperglycemia, a high glucose (HG) concentration of 35 mM was used. The HG medium was prepared by adding 29.5 mM D-glucose to the normal-glucose medium. Commercial osteogenic medium (Cyagen Biosciences Inc., USA) was used for osteogenic induction (OI). The Sep or Exo or Sep@Exo treatment were carried out simultaneously with the HG stimulation.

Cell viability

Cell viability was assessed using a cell counting kit (Dojindo, Japan) at the designated time point, following the instructions provided by the manufacturer. Briefly, the medium was discarded and replaced with 110 μL of CCK-8 working solution and incubated at 37 °C for 2 h. The OD values were detected by measuring the absorption at 450 nm wavelength as soon as possible.

Cellular immunofluorescence staining

All immunofluorescence staining experiments were conducted after 7 days of various treatments, unless otherwise specified. Cells were rinsed and fixed in 4% PFA, followed by permeation with 0.3% Triton X-100. After blocking with 5% BSA for 1 h, the cells were incubated with specific primary antibodies overnight at 4 °C. Subsequently, secondary antibodies or phalloidin were incubated for 1 h before performing DAPI staining for 10 min at room temperature. Images were captured using a confocal laser scanning microscope (Leica, Germany). Antibodies used in this study are listed in Table S1.

ALP staining and ALP activity assay

All ALP staining and ALP activity assays were conducted after 7 days of different treatments, unless otherwise specified. For ALP staining, cells were rinsed and fixed using 4% PFA. The BCIP/NBT Alkaline Phosphatase Color Development Kit (Beyotime, China) was used according to the manufacturer’s protocols and then samples were captured by scanner and microscope. ALP activity of MSCs was measured using an alkaline phosphatase assay kit (Beyotime, China). Cells were lysed with lysis buffer without inhibitors. The cell lysates were incubated with p-nitrophenyl phosphate (p-NPP) working solution (Beyotime, China) at 37 °C for 30 min, and then OD value at 405 nm wavelength was measured. Cell total protein concentration was detected using the BCA protein assay kit (Thermo Fisher Scientific, USA) for normalization of ALP activity.

Alizarin red S (ARS) staining

All ARS staining experiments were conducted after 21 days of different treatments, unless otherwise specified. Cells were fixed and rinsed with ddH2O. For mineralization observation, the fixed cells were stained with 1% ARS (Sigma-Aldrich, USA) and captured by scanner and microscope. For quantitative analysis, the samples were incubated with 10% cetylpyridinium chloride (Sigma-Aldrich, USA) at 37 °C for 60 min, OD value were measured at 590 nm wavelength.

RT-PCR

All RT-PCR detection were conducted after 7 days of different treatments, unless otherwise specified. The total RNA of the cells was isolated using RNAiso Plus (TaKaRa, Japan) and then reverse transcribed into complementary DNA (cDNA) using the PrimeScript 1st Strand cDNA Synthesis kit (TaKaRa, Japan). Real-time PCR reactions were performed using a Real-Time PCR system, the LightCycler® 480II (Roche, Switzerland). Glyceraldehyde-3-phosphate dehydrogenase (GAPDH) was detected to normalize mRNA expression levels. The relative gene expression levels

were analyzed using the $2^{-\Delta\Delta Ct}$ method. The PCR primer sequences used in this study are presented in Table S2.

Table S2 The primers for RT-PCR used in the present study

Gene name	Primer sequence (5’→3’)	Length
GAPDH	Forward: GACATCAAGAAGGTGGTGAAGC	22
	Reverse: TGTCATTGAGAGCAATGCCAGC	22
GRP78	Forward: CTGTGAGACACCTGACCGAC	20
	Reverse: CCGTGCCTACATCCTCCTTC	20
CHOP	Forward: AACCTGAGGAGAGAGTGTTCCA	22
	Reverse: CTGTCTCAAAGGCGAAAGGCA	21
PERK	Forward: ACAAGGCTGTCACTCAGGTG	20
	Reverse: GCTAGGAGCCTTGGAGCAC	19
GRP94	Forward: ACCGAAAAGGACTTGCGACT	20
	Reverse: TCAGCTCTCACAAACCCGAA	20
ATF6	Forward: CTCATGGACCAGGTGAAGACT	21
	Reverse: CCCAAGGCATCAAATCCAAATCA	23
ERN1	Forward: GCGCAGGTGCAATGACATAC	20
	Reverse: TCCACTTGATGGAGCCTGTC	20
Runx-2	Forward: CACAAGTGCGGTGCAAACTT	20
	Reverse: CTTGCAGCCTTAAATGACTCGG	22
ALP	Forward: CTCCTTAGGGCCACCGCTC	19
	Reverse: GAGATCCGTTCCTCGCTGGA	20
Col-1	Forward: CACTGCAAGAACAGCGTAGC	20
	Reverse: ACAAGCGTGCTGTAGGTGAA	20
OPN	Forward: GCTGAATTCTGAGGGACCAACT	22
	Reverse: CAAACTCAGCCACTTTCACCG	21
OCN	Forward: GAATAGACTCCGGCGCTACC	20

	Reverse: TCCTGGAAGCCAATGTGGTC	21
Shp2	Forward: ACGTCAGAGAAAGTGCTGCG	20
	Reverse: TTCCCTGGAGTAGAGCTTGTC	21

Extraction and purification of exosomes derived from MSCs

MSCs were initially cultured in α-MEM medium containing normal FBS. When the cell confluency reached 70-80%, the medium was replaced with exosome-free medium and harvested after 48 h. Then exosomes were isolated from the collected supernatant through a sequential centrifugation method, following a previously reported protocol.[3] Briefly, the medium was sequentially centrifuged at 300 g for 10 min, at 2000 g for 20 min, and at 10,000 g for 30 min at 4 °C to get rid of cells and debris. Next, the supernatant was filtered using a 0.22 μm filter (Merck Millipore, MA, USA) and then centrifuged at 100,000 × g for 90 min at 4 °C in a Type MLA-50 rotor using an Optima MAX-XP ultracentrifuge (Beckman Coulter, CA, USA) to harvest exosomes. The exosome pellet was washed in PBS and ultracentrifuged again at 100,000 g for 90 min. The resulting exosome pellet was then resuspended in PBS and stored at -80 °C for subsequent experiments. The protein concentration of exosomes was measured using the BCA protein assay kit (Thermo Fisher Scientific, USA).

Preparation of engineered exosomes loaded with Sep

Sep was engineered into exosomes through intermittent ultrasonic method. Briefly, 12 μg of Sep was mixed with 1 mg of exosome in 1 mL of PBS. The suspension was then sonicated intermittently and gently using a sonicator (20% amplitude, 10 s on/off, 2 min duration, 6 cycles, 2 min cooling period between each cycle). After being incubated at 37 °C for 2 h to restore the exosome membrane structure and form engineered Sep@Exo, the unloaded Sep molecules were removed using ultrafiltration tubes (Millipore, USA). The loading efficiency of Sep in exosomes was determined by calculating the amount of encapsulated Sep divided by the total input.

Morphology and size distribution of exosomes

For morphology analysis, the exosomes were fixed with 2.5% glutaraldehyde and placed on formvar-carbon-coated grids. They were then stained with a 1% uranyl acetate solution for 2 min. After being washed with distilled water and dried, the samples were captured using transmission electron microscope (FEI Talos L120C, USA) at 120 kV. The size distribution of exosomes was measured using the Nicomp 380 particle sizer (Z3000, PSS, USA).

Wetern blotting

Cell or exosome pellets were lysed with RIPA lysis buffer supplemented with protease inhibitors and ultrasonicated for 30 min on ice bath. Protein concentration was quantitated using the BCA protein assay kit. Western blotting was performed according to standard protocol. Briefly, the samples were separated through 4%-20% gradient SDS-PAGE gels and subsequently transferred onto polyvinylidene difluoride membranes (Millipore, USA). Next, the membrane was successively incubated with 5% BSA, specific primary antibodies, and HRP-conjucated secondary antibodies. After reacting with the ECL reagent, the signals were captured using the membrane imaging system (UVITEC, Britain). Antibodies used in this study are listed in Table S1.

Cellular internalization of exosomes

The exosomes were labeled with PKH26 dye (Sigma-Aldrich, USA) in accordance with the manufacturer’s instruction. After washing twice with DPBS buffer, PKH26-labeled exosomes were co-incubated with MSCs for 24 h at 37°C. Cells were rinsed and fixed, followed by staining with iFluor™ 488 phalloidin for 1 h and DAPI for 10 min at room temperature, and then captured with microscope.

EdU assay

The cell samples were collected after 4 days of different treatments, unless otherwise specified. The EdU Cell Proliferation Kit with Alexa Fluor 555 (Beyotime, China) was used to detect DNA synthesis and cell proliferation. The cells were incubated with 10 μM EdU solution for 2 h and washed with PBS buffer for twice. Then, the samples were fixed in 4% paraformaldehyde (PFA) for further staining with the Click Reaction solution and Hoechst 33342 (diluted in 1:1000) according to the manufacturer's protocol. The stained samples were visualized using a fluorescence microscope.

Flow Cytometry Analysis

The cell samples were collected after 7 days of different treatments, unless otherwise specified. The apoptosis rate of MSCs was analyzed by staining with the Annexin V-FITC apoptosis detection Kit (Beyotime, China) in accordance with the manufacturer's protocol. Briefly, cells were harvested, washed with PBS buffer, and then resuspended in 195 μL of binding buffer supplemented with 5 μL of Annexin V-FITC and 10 μL of PI staining solution. After incubating for 15 min at room temperature in the dark, all samples were washed and analyzed with Flow Cytometry.

Cell ultrastructure observation

The cell samples were collected after 7 days of different treatments, unless otherwise specified. Samples were harvested and fixed with 2.5% glutaraldehyde for 2 h at 4 °C, followed fixed with 1% osmic acid for 1 h at 4 °C. After fixation, the samples were dehydrated using a series of ethanol and acetone, and subsequently permeated and embedded in epoxy resin. The 70 nm ultrathin sections were stained with uranyl acetate and lead citrate and imaged using a transmission electron microscope (FEI Talos L120C, USA) at 120 kV.

Intracellular ROS measurement

The cell samples were collected after 7 days of different treatments, unless otherwise specified. The level of intracellular reactive oxygen species (ROS) was assessed using

the fluorescent probe DCFH-DA (Beyotime, China), following the manufacturer's protocol. Briefly, the cells were collected and rinsed with PBS before being exposed to a working solution of DCFH-DA at a concentration of 10 μM in a light-protected environment. Following a 30-minute incubation period and subsequent washing with PBS, the samples were observed and captured using a fluorescence microscope.

Co-localization of mitochondria and lysosomes

The cell samples were collected after 7 days of different treatments, unless otherwise specified. The cells were collected and rinsed with PBS, and then doublestained with Mito-Tracker Green (Beyotime, China) (100 nM) and Lyso-Tracker Red (Beyotime, China) (50 nM) for a duration of 30 min. Afterward, the cells were post-stained with Hoechst 33342 for 10 min. After being washed with PBS, the samples were visualized using a fluorescence microscope.

Mitochondrial membrane potential detection

The cell samples were collected after 7 days of different treatments, unless otherwise specified. The mitochondrial membrane potential of MSCs was determined by JC-1 assay kit (Yeasen, China). The cells were acquired, rinsed with cold PBS and then exposed to JC-1 working solution for a duration of 20 min at 37 °C in the dark. The cells were subsequently subjected to two washes with JC-1 buffer solution and then analyzed using flow cytometry.

Mitochondrial ROS detection

The cell samples were collected after 7 days of different treatments, unless otherwise specified. Mitochondrial ROS (mtROS) was detected using MitoSOX Red dye (Thermo Scientific, USA). The cells were rinsed with PBS, then stained with MitoSOX Red working solution for 30 min and Hochest 33342 for 10 min at 37 °C in order to detect the mtROS. Images were acquired using a fluorescence microscope.

Lentivirus transfection

To achieve a stable reduction in the expression of Shp2 in MSCs, Shp2-targeted shRNA and control lentivirus were designed and synthesized by Genomeditech (Shanghai, China). The targeting sequence of the shRNA was CCTCAACACAACTCGTATTAA. Lentivirus transfection was conducted according to the manufacturer's instructions. Briefly, MSCs were seeded in a 6-well plate and then incubated with lentiviral-shRNA at a multiplicity of infection (MOI) of 50, in the presence of 5 μg/mL polybrene. Positive cells were selected with puromycin (2 μg/mL). The transfection efficiency was detected by fluorescence microscopy and flow cytometry. The efficiency of Shp2 knockdown was confirmed by qPCR and western blotting analysis.

Synthesis of HA-A-C hydrogel

For the preparation of adipic acid dihydrazide grafted hyaluronic acid (HA-ADH), 1g HA (MW: 100~200 kDa) was dissolved in 200 mL of MES buffer (pH=5.3), then N-(3-Dimethylaminopropyl)-N'-ethylcarbodiimide hydrochloride (EDC, four molar equivalents of carboxyl group in HA) and 1-hydroxy-benzotriazole (HOBT, four molar equivalents of carboxyl group in HA) were added to activate HA for 2 h. Next, adipic acid dihydrazide (ADH, twenty molar equivalents of carboxyl group in HA) was supplemented to the mixed solution, pH was adjusted to 6.8 every 30 min for 4 h, followed by reaction for another 24 h. The reaction product was deeply dialyzed with NaCl (0.1 M) and deionized water in turn for purification. For the synthesis of aldehyde-modified hyaluronic acid (HA-CHO), 1 g HA (MW: 800~1500 kDa) was dissolved in 100 mL deionized water and then oxidized with sodium periodate (NaIO4, 0.025 M) in the dark. After 6 h, 1 mL ethylene glycol was supplemented to the mixture to stop the oxidation reaction process. The reaction product was purified by dialysis (10000 MWCO) with deionized water thoroughly in the dark. Afterwards, the purified HA-ADH and HA-CHO were lyophilized for subsequent use. The purified HA-ADH was characterized by 1H NMR (Bruker, Germany) in D_2O. The degree of hydrazide substitution was 32.50% determined by the proton peaks of hydrazide and HA. The

purified HA-CHO was characterized by fourier transformed infra-red (FTIR) (Thermo Fisher Scientific, USA). The oxidization degree of HA-CHO was 33.16% measured through the colorimetric hydroxylamine titration analysis. The ultimate HA-A-C hydrogel was prepared by mixing equal amounts of HA-ADH and HA-CHO in situ.

Gelling behavior

For gelling time determination, HA-A-C hydrogels prepared above with varying content were tested using the vial tilt method. Briefly, the hydrogel was added to a glass vial and left standing at 37°C for a period of time. When the gel solution stopped flowing after tilting the glass vial, the standing time was recorded.

Swelling behavior

For swelling behavior test, the HA-A-C hydrogels with different concentration were immersed in DPBS (pH = 7.4) at 37 °C for 24 h. The initial weight of the sample (W_0) and the weight of the sample after swelling (W_1) were recorded to calculate the swelling ratio using the following equation: Swelling ratio (%) = (W_1/W_0 - 1) $*$ 100%.

Degradation behavior

Briefly, the HA-A-C hydrogels were immersed in the PBS buffer for 24 h for swelling equilibrium. Then, the samples were incubated with PBS buffer containing 100 ng/mL of hyaluronidase to mimic the concentration of hyaluronidase in tissue, with PBS buffer serving as the control. The samples were placed in a constant temperature shaker and shaken at 60 rpm at 37 °C. The hyaluronidase buffer was renewed daily to maintain constant hyaluronidase activity. The remaining weight ratio (%) of the HA-A-C hydrogels against time point was recorded.

Rheology properties

The rheology properties of the HA-A-C hydrogel were tested on rotation rheometer (HAKKE MARS 60, Germany). Time sweep oscillatory tests of hydrogels with

different concentration were performed at 1 Hz and 1% strain (CD mode). For self-healing behavior characterization, strain sweep oscillatory test from 1% to 10,000% were performed on the HA-A-C hydrogel. The destroyed hydrogel was allowed to heal for 15 min, and then the self-healing effect was characterized by time sweep oscillatory test. The variation in viscosity of HA-A-C hydrogel measured with an alternate step shear rate being switched from small shear rate (0.1 s^{-1}) to large shear rate (500 s^{-1}). Each shear rate interval was kept as 120 s.

Macroscopic self-healing capacity

For macroscopic observation of self-healing capacity, hydrogels were gelled into tablet shapes and dyed with different colors. They were then cut into two parts and try to reconnect them after exchanges.

Lap shear tests

Fresh porcine skin (10mm × 10mm) was attached to an glass slide with cyanoacrylate glue. Since skin also contains amine groups and is easier to cut into uniform tissue slices, we used porcine skin to evaluate the adhesive strength of various hydrogel groups on tissues. A total of 60 μL hydrogel was uniformly dispersed on the surface of the porcine skin. A second porcine skin was attached to another glass slide and placed skin-to-skin on the first glass slide. After gelling in situ for 30 min at 37 °C, the bonded test samples were subjected for tensile mechanical test via a universal testing machine (Hengyi, China). The tissue adhesive strength of the hydrogel was determined according to the maximum pulling stress divided by the bonded area.

SEM observation

To observe the microstructures of the hydrogels, HA-A-C was lyophilized, followed by coating with gold-palladium. Next, the samples were captured by scanning electron microscopy (Zeiss, Germany). To evaluate the integration of the hydrogel and tissue, HA-A-C hydrogel was applied on pork tissue and gelling in situ. After lyophilization

and gold-palladium coating, the samples were observed through scanning electron microscopy.

Exosome distribution in hydrogel

The exosomes were first encapsulated in HA-A hydrogel solutions, then gelled with equal amount of HA-C solution for 15 min. Subsequently, the samples were visualized using confocal laser scanning microscope (Leica, Germany).

The exosome release profile of hydrogel

The equal amount of exosomes were added to HA-A-C gel and HAMA gel respectively. After gelation, the samples were soaked in a PBS buffer. 10 μL of PBS was collected at different check time points, and the same amount of fresh PBS was supplemented. The release profile of exosomes was detected by BCA protein assay kit.

The Sep release profile of hydrogel

The free Sep and engineereed Sep@Exo (containing equal amount of sep) were respectively supplemented to HA-A-C gel. After gelation, the samples were soaked in a PBS buffer and collected at the designated time points, and the release profile of Sep was measured by OD value at 290 nm wavelength.

The type 2 diabetic rat femur bone defect model

The type 2 diabetic rats were prepared according to a previous study.[4] Briefly, Four-week-old male Sprague Dawley (SD) rats were treated with high fat diet for 4 weeks. Then the rats were received intraperitoneal injection with streptozotocin (STZ) at a dose of 30 mg/kg. One week later, the rats with blood glucose levels consistently higher than 16.7 mM were considered diabetic. Subsequently, the the diabetic rats were randomly divided into 4 groups: 1) Blank group, 2) Gel group, 3) Gel-Exo group, and 4) Gel-Sep@Exo group. For specific animal surgical procedures, the operation site were shaved and disinfected after general anesthesia (sodium pentobarbital 30 mg/kg). A

defect with a diameter of 3 mm and depth of 2.5 mm was prepared near the medial epicondyle of each rat with a hand drill. Then different groups of hydrogel were injected to fully fill the defects, and the surgery sites were sutured layer by layer. Each group is in sextuplicate.

Micro-CT analysis and histological evaluation

Half of the rats were sacrificed at the fourth week and the other half at the eighth week. The femurs were extracted and fixed in formalin. Micro-CT (μCT) analysis of fixed samples was performed using a μCT50 (Scanco Medical, Switzerland) at 70 kVp, 114 μA and a resolution of 15 μm. The defect area was selected as the region of interest (ROI) for quantification of bone volume/tissue volume (BV/TV), bone mineral density (BMD), trabecular number (Tb.N) , trabecular thickness (Tb.Th) and trabecular separation (Tb.Sp). Later, the specimens were decalcified with 20% EDTA for one month and then embedded in paraffin to be sliced into 4 μm for subsequent histological evaluation. Histological analysis and immunofluorescence staining of bone tissue were conducted following the aforementioned steps. To examine the biosafety of the Gel/Sep@Exo, major organs including heart, liver, spleen, lung and kidney of different groups were histologically evaluated after implantation for 8 weeks.

Statistical analysis

All data are presented as mean (SD). Statistical analyses were conducted using GraphPad Prism 9 statistical software (GraphPad, USA). Group differences were assessed using either Student's t-test or one-way analysis of variance (ANOVA). The threshold for determining statistical significance was established at a significance level of $P < 0.05$. The symbols *, **, and *** are used to denote P values less than 0.05, 0.01, and 0.001, respectively, while "n.s." indicates not significant.

Supplementary Figures

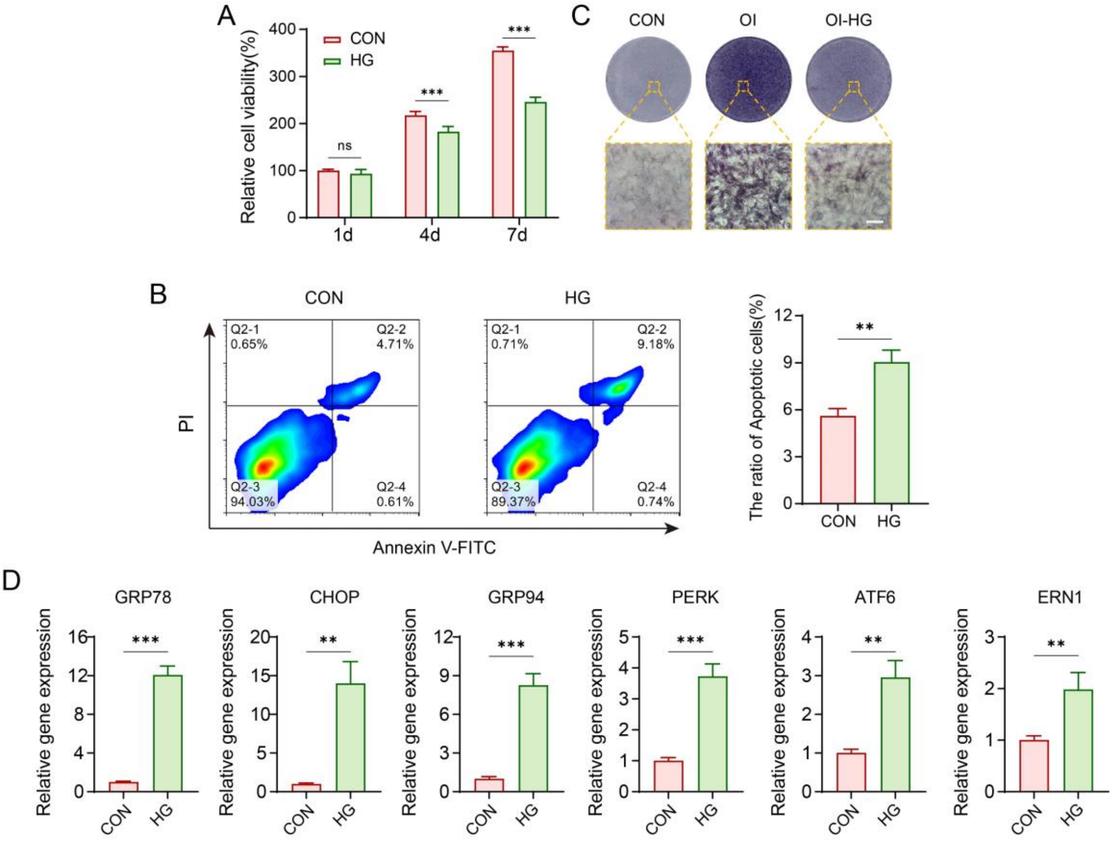

Figure S1. Comparison of cell viability (A), osteogenic differentiation (B), cell apoptosis levels (C) and the expression of ER dysfunction-related genes (D) in MSCs cultivated in HG medium with the normal control group (n=3). Scale bar: 100 μm.

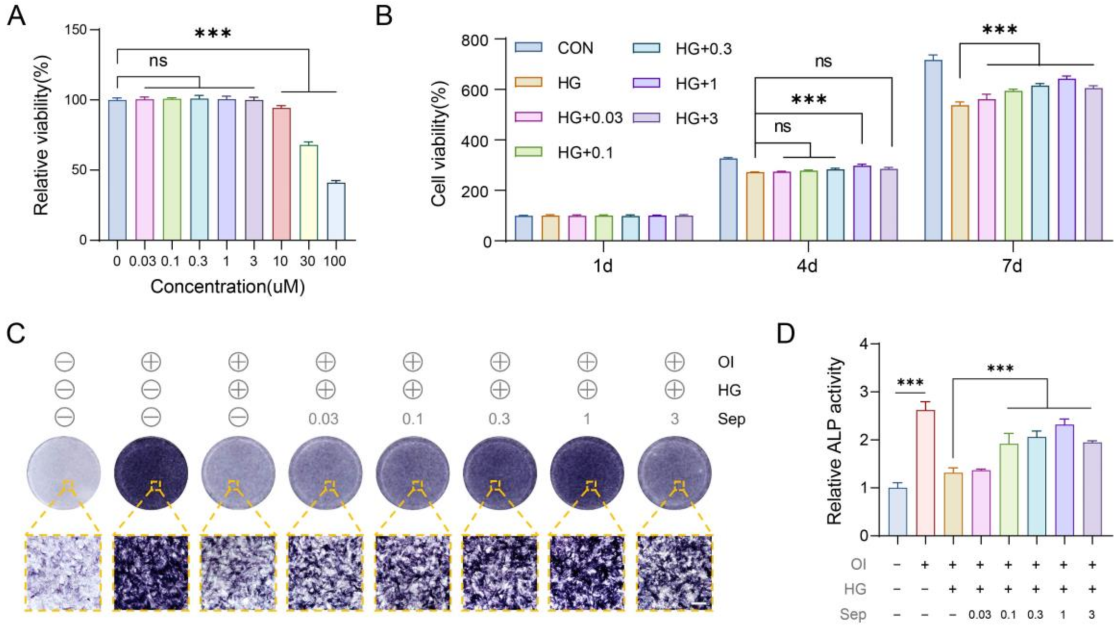

Figure S2. A) Cell viability of MSCs cultured with various concentrations of Sep for 24 h in normal glucose medium (n=5). B) Cell viability of MSCs cultured with various concentrations of Sep in HG medium at different time point (1 d, 4 d, and 7 d) (n=5). C) ALP staining and D) ALP activity semiquantitative analysis of MSCs cultured with various concentrations of Sep for 7 days in HG medium (n=3). Scale bar: 100 μm.

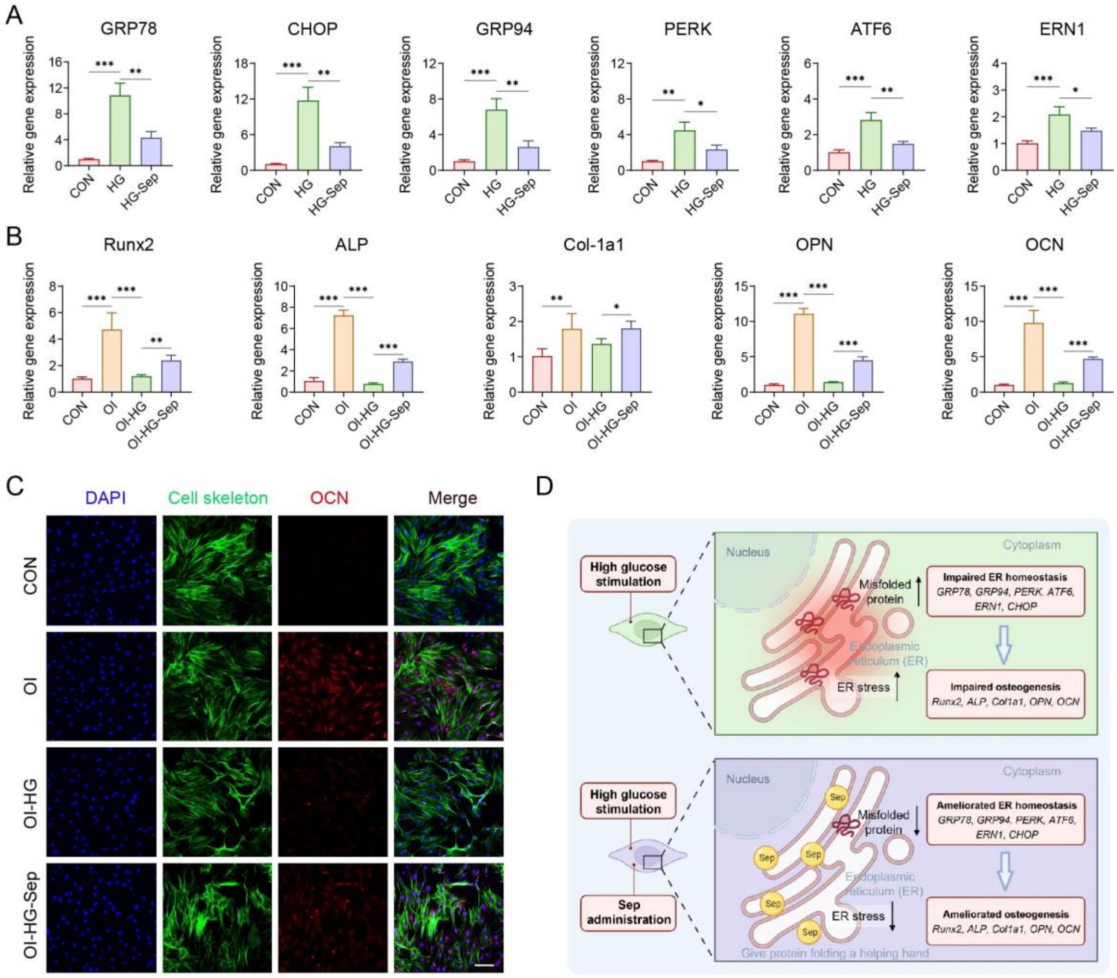

Figure S3. A) Endoplasmic reticulum dysfunction related genes' expression of MSCs with or without Sep administration (n=3). B) Osteogenesis-related genes' expression of MSCs with or without Sep administration (n=4). C) Immuno-fluorescence staining of OCN in MSCs with or without Sep administration (n=3). Scale bar: 100 μm. D) Schematic illustration describes that Sep administration ameliorates ER homeostasis and facilitates osteogenesis of MSCs in HG microenvironment (created with BioRender.com).

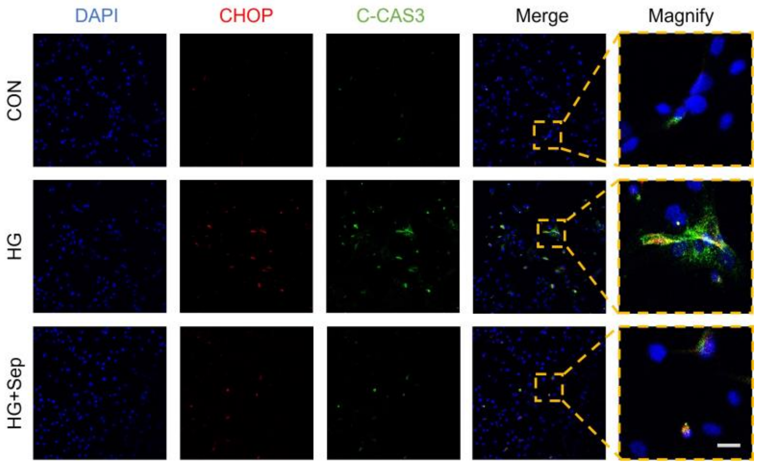

Figure S4. Co-immunofluorescence staining of C-CAS3 and CHOP in MSCs cultivated in HG medium with or without Sep administration for 7 days (n=3). Scale bar: 20 μm.

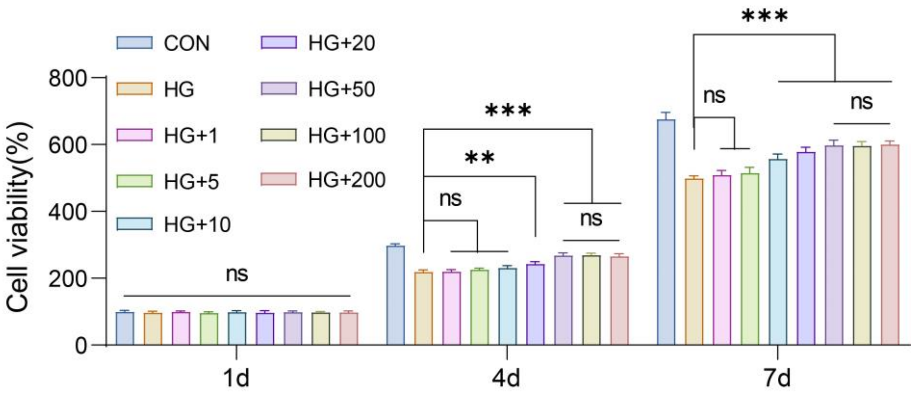

Figure S5. Cell viability of MSCs was assessed after culturing with various concentrations of exosomes, ranging from 1 μg/mL to 200 μg/mL, in HG medium at different time points (1 d, 4 d, and 7 d) (n=5).

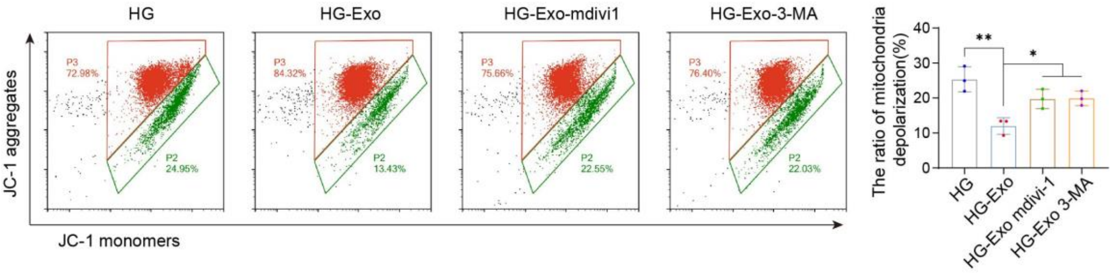

Figure S6. Flow cytometry detected JC-1-mitochondria membrane potential of MSCs treated with exosomes with or without mitopghagy inhibitors in HG microenvironment (n=3).

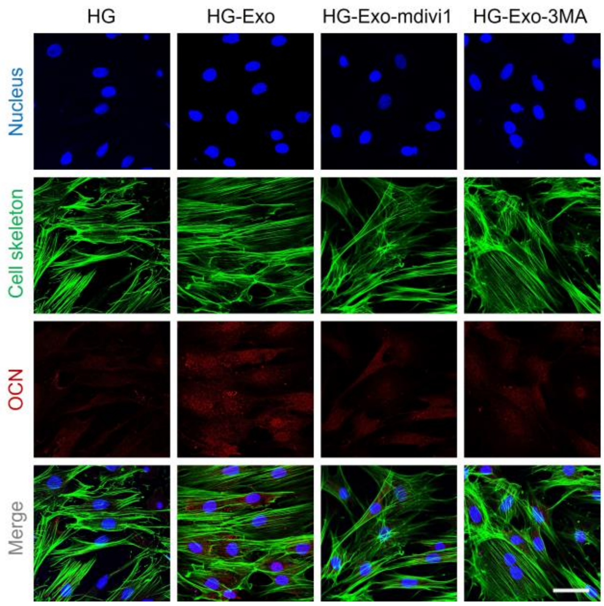

Figure S7. OCN immunofluorescence staining of MSCs treated with exosomes with or without mitopghagy inhibitors in HG microenvironment (n=3). Scale bar: 50 µm.

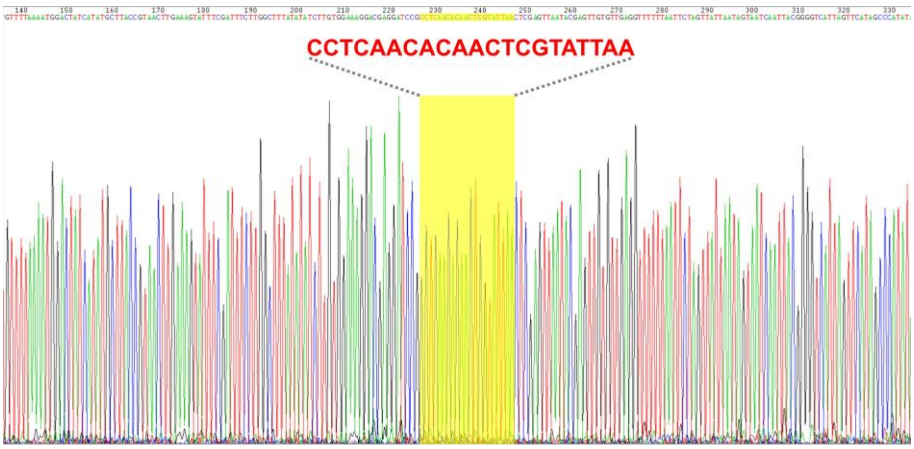

Figure S8. The successful construction of the Rat_Shp2-shRNA interference vector

was confirmed through the analysis of the sequencing results.

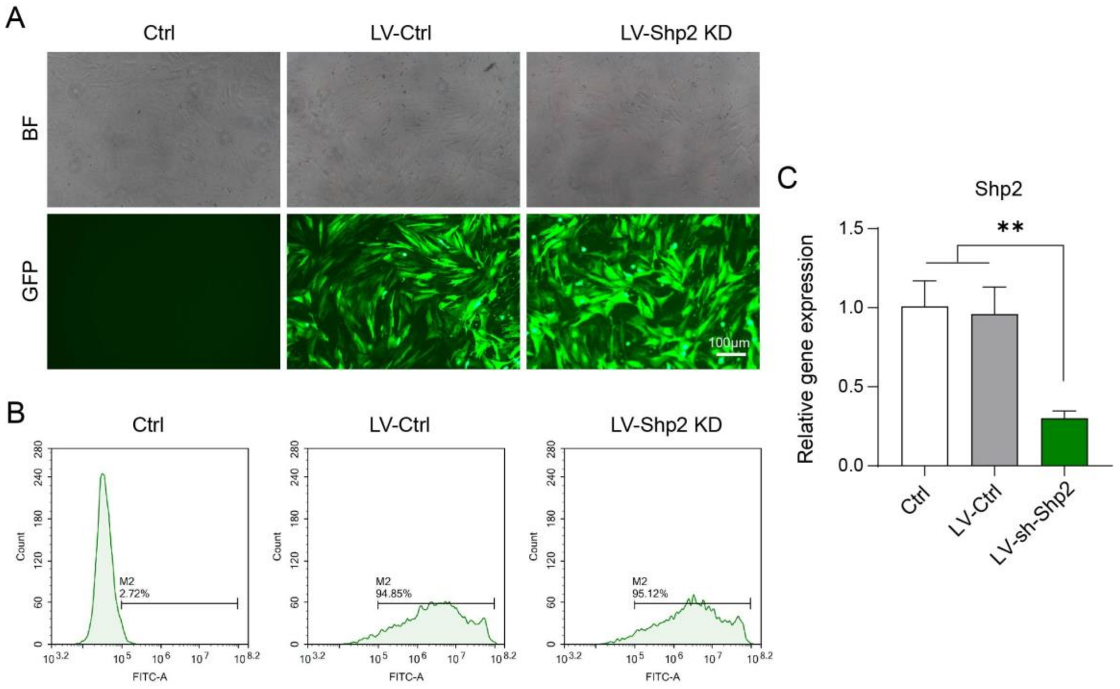

Figure S9. MSCs were transfected with non-specific lentivirus vector (LV-Control) or Shp2-targeted shRNA lentivirus (LV-Shp2 KD). The transfection efficacy was obeserved by (A) fluorescence microscopy and measured by (B) flow cytometry. Over 90% of the transfected cells appeared positive. (C) RT-PCR were performed to confirm the mRNA level of Shp2. GFP: green fluorescent protein (n=3).

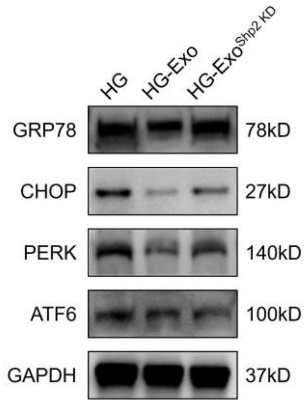

Figure S10. Western blot detected the expression of ER dysfunction-related proteins in MSCs treated with Exo or Exo$^{Shp2\ KD}$ in HG microenvironment.

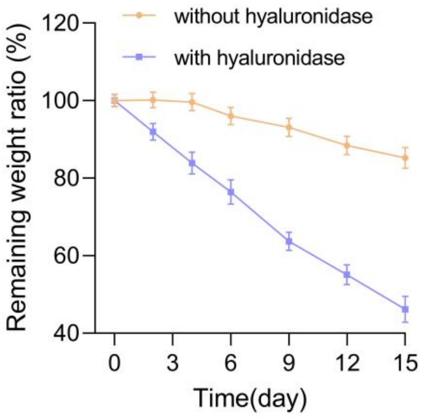

Figure S11. The remaining weight ratio against degradation time for the HA-A-C hydrogel in the presence or absence of hyaluronidase (100 ng/mL).

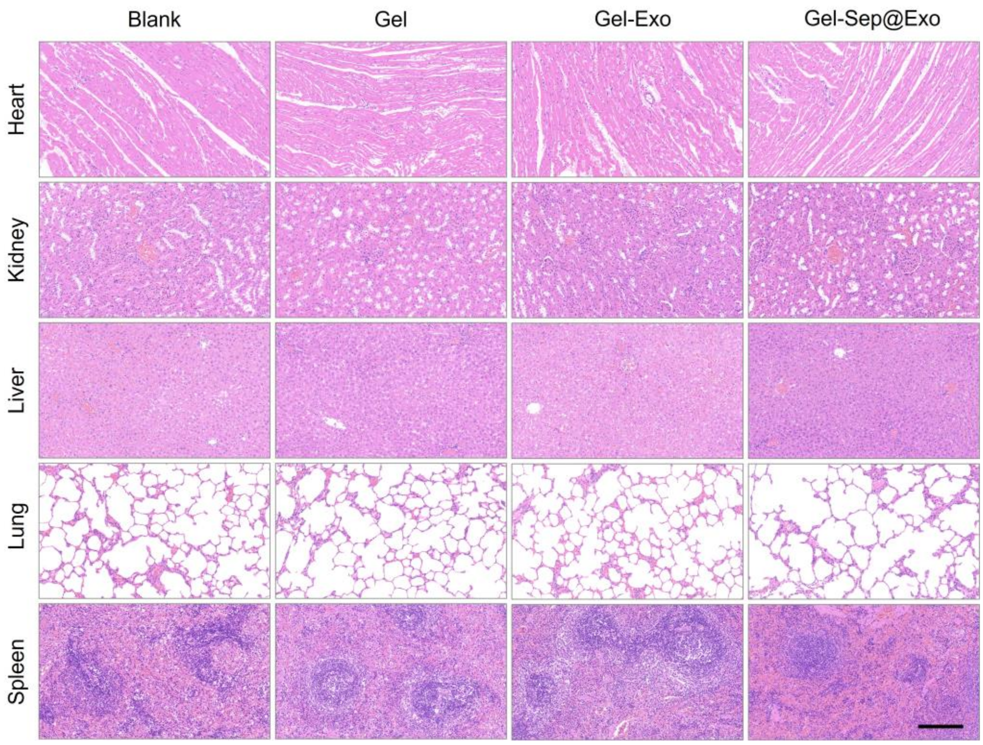

Figure S12. The H&E staining images of metabolic organs indicated excellent in vivo biocompatibility of the hydrogel and Sep@Exo (n=3). Scale bar: 200 μm.

Reference

[1] L. Edgar, N. Akbar, A.T. Braithwaite, T. Krausgruber, H. Gallart-Ayala, J. Bailey, A.L. Corbin, T.E. Khoyratty, J.T. Chai, M. Alkhalil, A.F. Rendeiro, K. Ziberna, R. Arya, T.J. Cahill, C. Bock, J. Laurencikiene, M.J. Crabtree, M.E. Lemieux, N.P. Riksen, M.G. Netea, C.E. Wheelock, K.M. Channon, M. Ryden, I.A. Udalova, R. Carnicer, R.P. Choudhury, Circulation, **2021**, 144, 961-982.

[2] S.H. Lin, S. Yin, J.F. Shi, G.Z. Yang, X.T. Wen, W.J. Zhang, M.L. Zhou, X.Q. Jiang, Bioact. Mater., **2022**, 18, 116-127.

[3] C. Thery, S. Amigorena, G. Raposo, A. Clayton, Current protocols in cell biology, **2006**, Chapter 3, Unit 3.22-Unit 23.22.

[4] C. Yang, Z.W. Zheng, M.R. Younis, C.L. Dong, Y.H. Chen, S. Lei, D.Y. Zhang, J.Y.Z. Wu, X.Q. Wu, J. Lin, X.S. Wang, P. Huang, Adv. Funct. Mater., **2021**, 31.
